# Anomaly detection in genomic catalogues using unsupervised multi-view autoencoders

**DOI:** 10.1186/s12859-021-04359-2

**Published:** 2021-09-25

**Authors:** Quentin Ferré, Jeanne Chèneby, Denis Puthier, Cécile Capponi, Benoît Ballester

**Affiliations:** 1 INSERM, TAGC, Aix Marseille University, Marseille, France; 2 Université de Toulon, CNRS, LIS, Aix Marseille University, Marseille, France

**Keywords:** Genomic assay, Anomaly detection, Cis regulatory element, Unsupervised curation, Convolutional autoencoder, ChIP-seq peak quality, Model interpretability

## Abstract

**Background:**

Accurate identification of Transcriptional Regulator binding locations is essential for analysis of genomic regions, including Cis Regulatory Elements. The customary NGS approaches, predominantly ChIP-Seq, can be obscured by data anomalies and biases which are difficult to detect without supervision.

**Results:**

Here, we develop a method to leverage the usual combinations between many experimental series to mark such atypical peaks. We use deep learning to perform a lossy compression of the genomic regions’ representations with multiview convolutions. Using artificial data, we show that our method correctly identifies groups of correlating series and evaluates CRE according to group completeness. It is then applied to the ReMap database’s large volume of curated ChIP-seq data. We show that peaks lacking known biological correlators are singled out and less confirmed in real data. We propose normalization approaches useful in interpreting black-box models.

**Conclusion:**

Our approach detects peaks that are less corroborated than average. It can be extended to other similar problems, and can be interpreted to identify correlation groups. It is implemented in an open-source tool called atyPeak.

**Supplementary Information:**

The online version contains supplementary material available at 10.1186/s12859-021-04359-2.

## Background

The decreasing cost of gene sequencing and other genomic assays localizing various regions of interest (epigenomic features, TF binding regions) has resulted in a wealth of experimental data from the broader scientific community as well as from large consortia (e.g., ENCODE [1]). This data has been collated in warehouses such as the GEO database [2] or ArrayExpress [3] to facilitate inference and functional annotation of genomic regions. This includes Cis Regulatory Modules (CRMs), which regulate gene expression through binding Transcriptional Regulators (TRs), including Transcription Factors (TFs) binding directly to DNA, and co-factors binding to other TRs forming a regulator complex. Localized clusters of TR bindings form Cis-Regulatory Elements (CREs). In this paper, we focus on improving CREs detection and characterization through better identification of TR binding locations.

While TF binding sites (TFBS) may be predicted based on DNA sequence, statistical precision is low [4] and the use of experimental data, such as ChIP-seq [5] that combines chromatin immunoprecipitation with massively parallel DNA sequencing, is preferred [6]. ChIP-seq can detect both TFs and co-factors binding regions. Each region is associated to a peak in the signal, wider than the region. However, such large scale NGS approaches are known to contain errors and biases resulting in artifactual or anomalous elements. Low complexity regions complicate mapping [7], and ChIP-seq presents several known difficulties [8] including immunoprecipitation quality [9] and poor antibody specificity, inadequate experimental controls, sequencing biases (mostly uneven sonication), and other factors complicating peak calling [10]. False positives can be introduced for biological reasons [11, 12] and through peak callers (FDR of 1–5% or more [13]). Besires errors, anomalous peaks can be caused by other biological and technical specificities (e.g., different protein fixation kinetics), systematic experimentator biases, mutations creating new TFBS, TRs having rare secondary roles, etc.

Such problems are difficult to correct a posteriori. In some cases, human manual curation is possible to label artifacts that can be subsequently used to train supervised machine learning models, some of which also leverage deep learning and combinations between series [14]. But this is seldom available. There is currently no systematic detection method or database of known false-positive regions, besides the ENCODE blacklist [15]. To mitigate this, one can enforce quality criteria at each step, from sequencing (read quality, existence of replicates) to mapping (proportion of mapped reads, low number of regions mapped by unique reads) to peak calling (IDR). The IDR [16] measures consistency between peaks called for two replicas of the same biological condition. However, it is only pairwise, singles out entire series and not individual peaks, and if one of the two replicas considered has poor quality both replicas will get a low score. It also cannot be applied to corroborate data from two different protocols or conditions (e.g., different laboratories or TRs).

While quality criteria can be computed for every step of the data processing, which ENCODE does, large amounts of data nevertheless increase the risk of at least one false positive being present and would be detrimental to CRE analysis. Here, we seek to work at a higher scale and use correlations between processed data. As supervised curation is unavailable, the question is whether we could use another property of the data to identify inconsistent elements. Since TRs most often act in combination through complexe formation [6], it follows that biologically significant CREs would likely be clusters of TRs. Similarly, different experimental series should have patterns of corroboration (e.g. where series A says there is a peak, series B says so as well). Here, we define a dataset as one ChIP-seq experimental series for a given TR (in a given cell line, those can be technical replicates or different experiments). For example, RAD21 is significantly associated with CTCF in insulator regions [17]. As such, RAD21 and CTCF form a correlation group and finding CTCF alone would be suspicious. Such combinatorics are indeed considered of major interest to CREs detection [18] and meta-analysis of datasets is emerging [19]. As such, we turn to the more general problem of anomaly detection, meaning to identify elements that do not conform to the expected normal patterns [20], as a substitute for curation. In this study we focus on detecting anomalous or atypical peaks, where atypical is defined as not respecting the typical TR and/or dataset combinations learned from the data. Removing anomalous peaks will, in turn, fulfill our objective of improving CRE quality. As CREs have high peak density, anomaly detection methods can be used.

We consider the ReMap [21] database, whose initial curation and uniformized reprocessing workflow provide sufficient quantity and quality to use the outlier detection approaches we propose. Given the volume of data and the potential complexity of the combinations, Deep Neural Networks (DNN) models are a natural solution and have been used before on genomic data [22]. They are able to learn complex distributions, not achieved by methods such as PCA, by using multiple layers of increasingly-abstracted representations. Specifically, autoencoders are known to be effective in unsupervised anomaly detection. They also allow us to work at the level of individual loci. Furthemore, this is also a multiview problem [23] as each TR can be thought of as one view composed of several datasets. Thus, our approach integrates correlations between datasets and/or TRs, leveraging another strength of NNs.

To remove atypical peaks, we propose atyPeak, a stacked convolutional autoencoder, and supply processed data files for selected TFs and cell lines from ReMap. Since no gold standard dataset exists to perform cross-validation we demonstrate our approach with artificial data to ensure robustness of our model [24]. Our approach is applicable to any series of intervals, not only ChIP-seq regions. It also offers some interpretability and can be used to extract and interpret the aforementioned correlation groups, as identification of clusters of TRs is of great interest [25].

## Results

### Representation and processing of cis regulatory modules

**Fig. 1:**
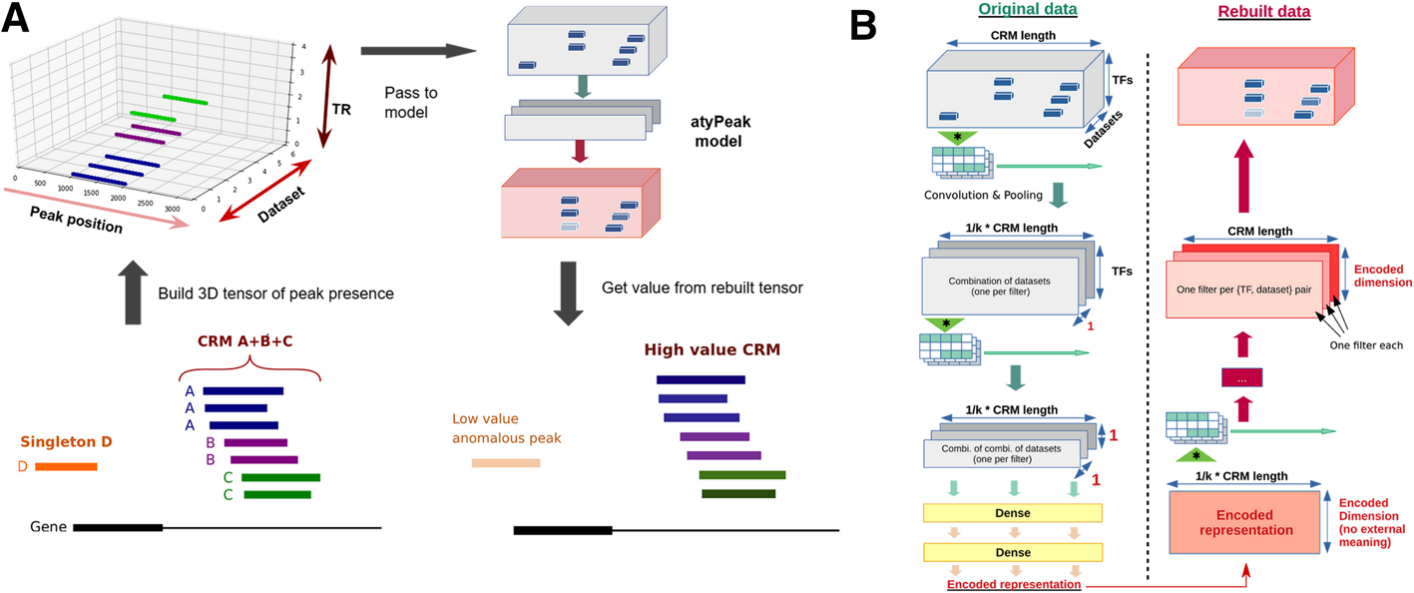
**a** Workflow and model description. Once candidate regions (ReMap-identified CRMs) are set, we build tensors of peak presence representing them. The X axis represents the position along the genome, while the Y and Z axis are dataset and TR identifiers respectively. The tensor has a value of 1 is a peak for this TF in this dataset (i.e., for this source) is present, 0 otherwise. The atyPeak model will lossily compress this representation. This will result in losing anomalies and other finer details, by learning correlation groups for the rebuilding instead of individual peaks. At the end, each peak is given an anomaly score corresponding to the mean autoencoder reconstruction error, the difference between the original (grey) and rebuilt (red) representation. Scores are then added to the original BED file. Full source code and documentation are available at < https://github.com/qferre/atypeak > . **b** Model structure. During the encoding, the CRM are viewed by the model through convolutional filters to focus on the correlations between datasets and then between TRs. We use two type of filters (combinations of datasets, then combinations of TRs) successively in a stacked multiview approach. After the subsequent Dense layers, we obtain a smaller encoded representation. This encoded representation is fed to a convolutional decoder with several layers, trying to rebuild the original CRM representation. In subsequent figure legends, “deep dimension” is the number of neurons in each Dense layer, while the “filters number” is the number of kernels in each Convolutional layer, and LR is the learning rate of the Adam optimizer. More details about the structure are available in Methods

To apply our method, each CRM is first converted to a 3D tensor representation of the peaks it contains, where the X, Y, Z axes represent respectively genomic position, datasets of origin, and TR of interest (Fig. 1a). We then use a convolutional autoencoder to perform a lossy compression. The representations are viewed by the model through convolutional filters. They focus first on the correlations between datasets and then between TRs, in a stacked multiview approach (Fig. 1b). This produces an encoded representation of the CRM, passed to a decoder attempting to reconstruct the original. In the end, each peak is given an anomaly score corresponding to its difference in value in the original and rebuilt representations.

Our approach can be applied to any type of sets-of-intervals data from multiple sources in the same format, not only omics. In subsequent figure legends, “deep dimension” is the number of neurons in each Dense layer, while the “filters number” is the number of kernels in each Convolutional layer. LR stands for learning rate.

### Validation using artificially generated data

Without gold-standard data, and in the absence of precedent readily available methods that are comparable to our approach (cf. Introduction and Methods), we generate artificial data designed to approximate real CRMs to confirm the model’s ability to correctly discover correlation groups of sources (a peak’s source is its {TR, dataset} pair). Our goal is to simulate biological complexes of TRs, each one being a correlation group. To generate artificial regions, we stack a random number of peaks around a given position. The sources of those peaks belong to one of two (or more) predefined sets of sources representing correlation groups (Additional file 1: Fig. S1). The choice of set is made once per CRM. As a result, peaks from each set will significantly correlate only with other members of the same set, forming a correlation group. We also add to the CRM random noise representing atypical peaks not respecting existing correlation groups.

**Fig. 2:**
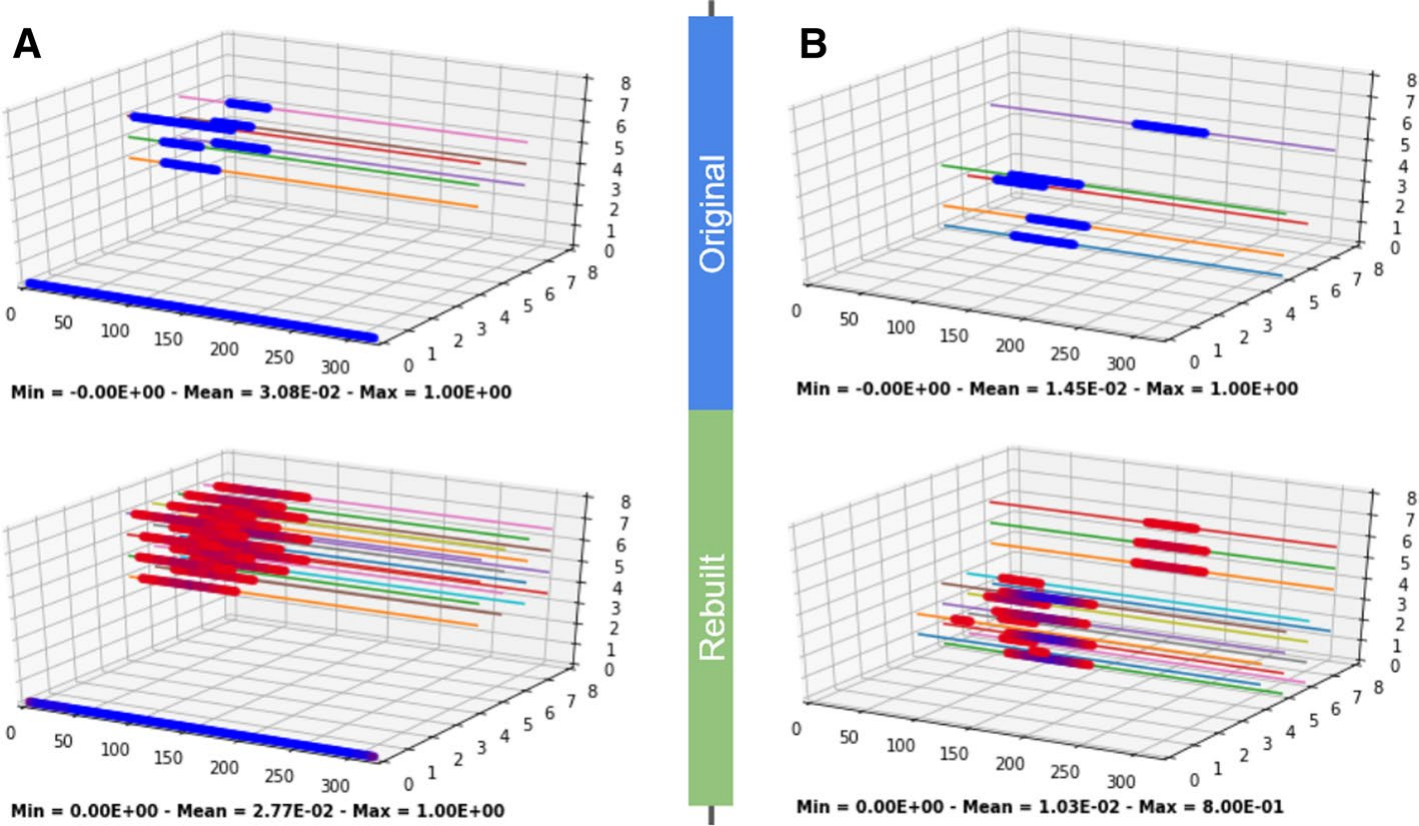
The atyPeak model learns correlation groups. In each case, the tensor at top is the original representation and the bottom one is what is rebuilt by the model. The model was trained on artificial data. There were 2 predefined correlation groups covering different subsets of dimensions (G1 and G2) defined in Additional file 2: Fig. S2. The thin colored lines are only here as a visual aid. In **a** when the model rebuilds the CRM representation, it rebuilds the entire correlation group when peaks from the group are present. This results in adding the other members that were not originally present as “phantom” peaks. In this case, it is the G1 group. In **b** however, we used a model with a less aggressive compression (too high information budget) and the rebuilding is too precise, learning smaller, non-significant groups instead of the entire G1 or G2 groups. Model parameters in **a** were a deep dimension of 32, 16 filters and a Learning Rate (LR) of 1E−3. **b** Used 48 filters, 256 deep dimension, a LR of 1E−4. Note that for B, that increased precision is not achieved with higher deep dim but default LR—we needed a lower LR. 48 epochs for all or early stopping (for **a**). Groups were equiprobable

We tested our model’s ability to learn which predefined correlation group each peak belongs to, as opposed to the individual peak itself. Indeed, when peaks from a given correlation group are present, the model rebuilds the entire group in the neighborhood of the peaks and not the individual peaks (Fig. 2a, 2 groups). A peak’s value in the rebuilding depends on how many sources from its group are present. The more complete the group is, the higher the value (although fully complete groups are unlikely to occur in the actual data). Peaks added to the rebuilt tensor by the model are called phantoms (Fig. 2). Biologically, this means that the model will identify common TRs and/or dataset combinations. Each peak will get a score proportional to the number of correlators present in its vicinity, and the missing correlators will be added as phantoms.

#### Stability to different group characteristics

Our model is still effective with more complex and realistic artificial data models. For instance, the number of binding sites across TRs differ but we show that the model is not biased by differences in abundances between correlation groups (Additional file 3: Fig. S3a). Furthermore, biological correlation groups are not mutually exclusive (e.g., for the TRs A, B and C, there can be both an “ABC” and an “AB” group) and the model is also capable of learning such groups (Additional file 4: Fig. S4B and Additional file 5: Fig. S11) although this is not always reliable and comes with caveats and precautions described in Methods. Generally, phantom peaks from overlapping groups will be generally present but less pronounced than they should be.

It is possible to extract the correlation groups learned by the model to mine for biologically relevant combinations of sources. It can be done by interpreting the encoded dimension (Additional file 6: Fig. S6), or instead by identifying the correlators of a given source by looking at which phantoms peaks are added when it is present (Fig. 2, and Additional file 5: Fig. S11 in real data).

#### Scalability and the information budget

Any compression is characterized by its aggressiveness: one that is not aggressive enough can afford to learn details (for us, the noise we want to remove) but a too aggressive compression might lose too much information. In our method, this depends on the relative information budget, which is the ratio between the data’s information and the model’s entropic capacity. Biologically, this means the budget to be used depends on the size of the database: the number of sources considered and the number of relevant correlation groups in the data. For example, in Fig. 2b the model with a too large budget learns too precise, smaller, non-significant correlation groups but the phantom peaks added are still not from sources outside the correlation group.

**Fig. 3:**
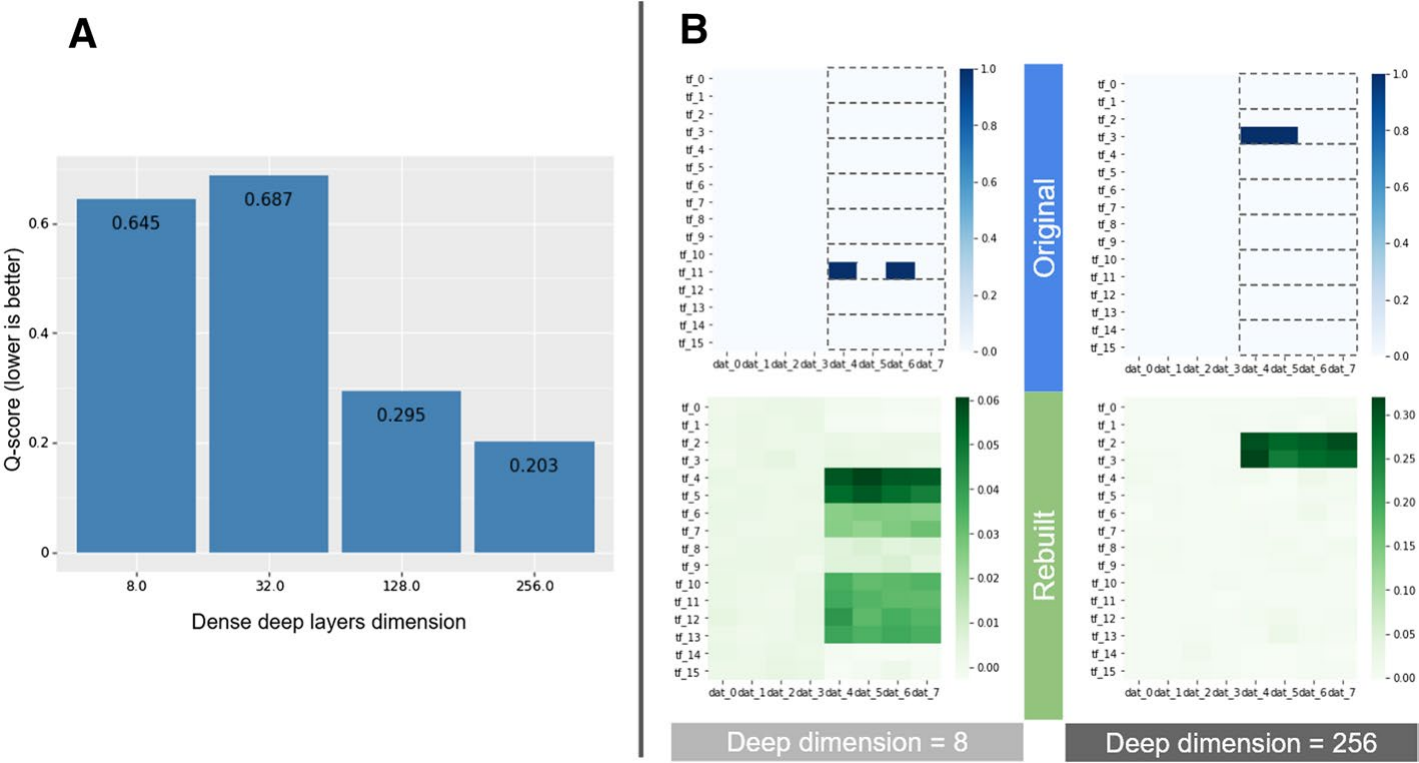
Scaling of the information budget with the data. We used artificial data of dimension 8 × 16, but the TRs are subdivided into 8 groups instead of 2 like in Additional file 2: Fig. S2 (I.e., there the TR 0 and 1 are a group, then 2 and 3 are another group, etc. up to 14 and 15. The groups are visually reminded on the figure as one grey box per group). At data generation, the stack is placed in one of the 8 groups. All 8 groups are equiprobable. The model parameters were 24 convolutional filters and a LR of 1E−4. The number of neurons in the Dense layers changes during the grid search. With lower deep dimensions (and so a lower information budget), the model is unable to learn separately the 8 existing correlation groups (B left) and will instead learn fewer and larger groups. A larger budget was needed to learn the 8 groups (B right). This highlights how the information budget must be adapted to the quantity of information in the data for a satisfactory result. Note that for this larger data, hundreds of neurons are required, compare to smaller models for the smaller data of Fig. 2. To help choose the budget, we propose a Q-score to quantify the quality of the rebuilding depending on the budget. This score assesses how well the model learns each existing pairwise correlations. More details about the Q-score of the models involved in this figure is presented in Additional file 7: Fig. S5

To rigorously choose the information budget, we propose to verify that the model correctly learns pairwise correlations. We design and propose a quality score (Q-score) which is also used on real data. In Fig. 3, we demonstrate scaling budget upwards to accommodate richer data with 8 correlation groups. The model tends to focus on the most frequent sources when learning the groups, which can often result in grouping the least sources together in background groups and ignoring the very rarest ones. These differences in focus can be alleviated (Additional file 4: Fig. S4a), but mean that a correlation group does not necessarily represent a single or complete biological complex, which should be remembered when interpreting them.

#### Systematization on many observations

In Additional file 1: Fig. S1, we show that the observations made above hold true when considering a larger number of artificial CRMs. The presence of a peak results in phantoms peaks from the same correlation group of sources, but not from the other groups. Noise peaks, lacking their usual correlators, get rebuilt with a lower value. Models with a too large budget will learn smaller non-significant groups. atyPeak is still robust when the noise represents a higher proportion of the signal but does not overwhelm it (Additional file 1: Fig S1B), but this will result in noisy peaks gaining a comparatively higher score, as in other unsupervised approaches.

The learned group themselves can be subject to certain biases, such as giving higher values to comparatively more abundant elements within a group, the fact that all correlation groups do not have the same average completeness, and contributions to the rebuilt values from several overlapping groups. We performed normalization to counter such biases, by evaluating the score given by the model in controlled artificial CRMs when only peaks from the considered source are present, and normalizing by TR in the end. Non-normalized results are also available, but the score must then be interpreted relative to the average score for its source. More details about the experiments conducted on artificial data and the conclusions drawn are available in Methods.

### Application to real biological data

Having evaluated our model on artificial data, we processed biological data from the ReMap project. We used selected CRMs for the Jurkat, Hela, K562, MCF7, CD34 and ESC cell lines. This data was sparser than the artificial datasets, as not every dataset contained every TR. This necessitated “crumbing” (Additional file 8: Fig. S9) and adapting the information budget. Parameters are detailed in Additional file 9: Table S2 and were chosen thanks to the Q-score. We covered a variety of cell line profiles, ranging from Jurkat’s sparse genomic binding data with many datasets concerning only one TR, to high-dimensionality examples such as K562 proving our model’s scalability potential.

**Fig. 4:**
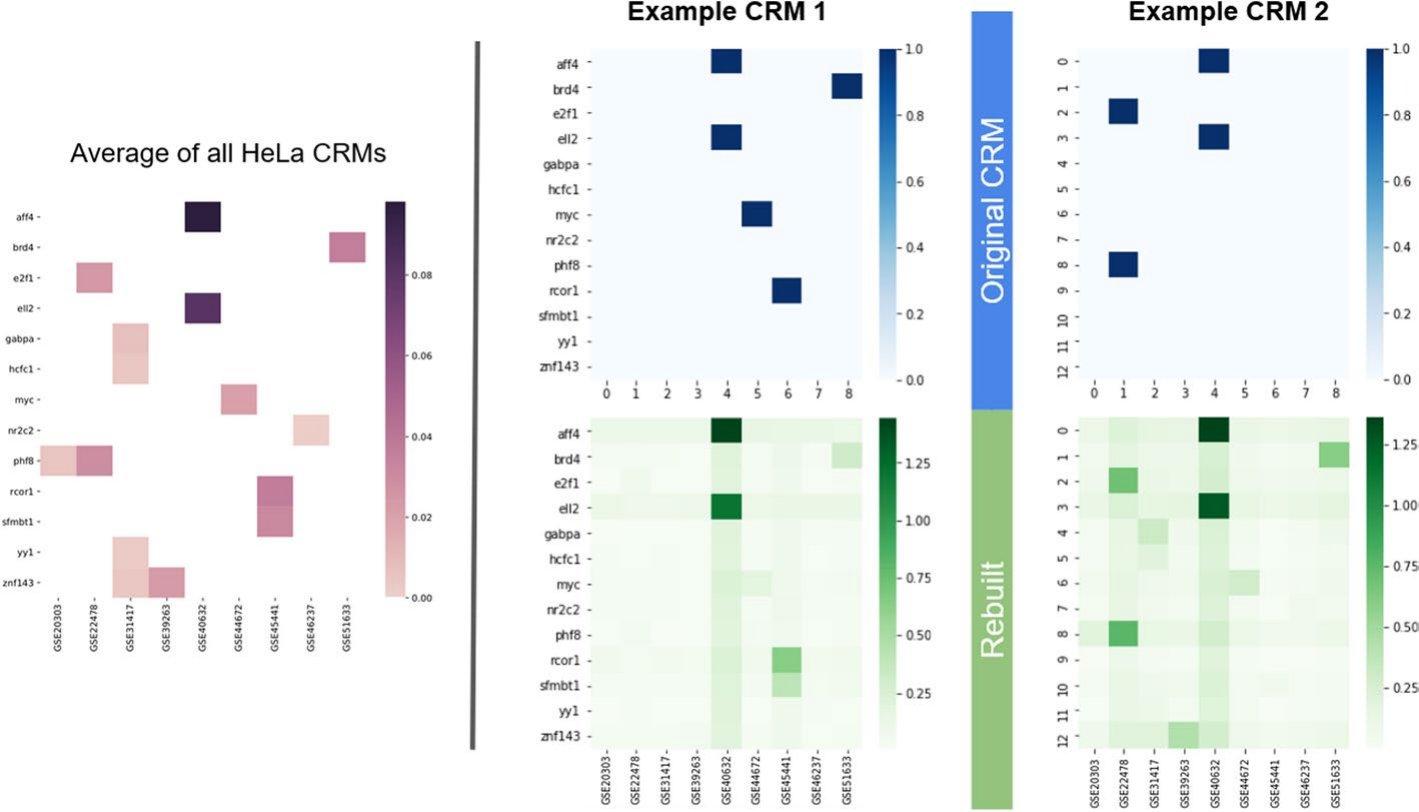
Two different examples of real CRMs rebuilt in HeLa. All figures give the mean across X axis (region size) of the tensors. The model’s parameters are detailed in Additional file 10: Table S1. As with the other figures, the blue heatmap represents the original representation of the CRM, with the green heatmap giving the rebuilding by the model. The average of all HeLa CRMs is provided for comparison. The model has visibly learned different correlation groups, and not just rebuilt the average CRM. We can see, notably for BRD4, that an incomplete group results in lower scores, and that phantoms are added to complete the learned groups. Some learned groups are extracted and presented in Additional file 5: Fig. S11 for comparison

Figure 4 shows two representative examples of CRMs in HeLa along with their rebuilding by the model. The difference in rebuilding shows that the model does not always rebuild the average CRM and has learned different correlation groups. In Fig. 4a, BRD4 has a low score as the model was expecting more correlators (cf. the estimated correlation groups presented in Additional file 5: Fig. S11), unlike AFF4 and ELL2. Conversely, in Fig. 4b the BRD4 peak was expected and is added as a phantom.

In HeLa, we observed 3–4 different correlation groups learned by the model, in line with what is likely biologically significant. In practice more groups are learned as overlaps of groups: this is visible in HeLa with AFF4 and ELL2 still benefiting from the presence of other sources (Additional file 11: Fig. S8), and BRD4 or SFMBT1 being part of several correlation groups (Additional file 5: Fig. S11). Across most cell lines, the model usually needs to learn over only a few thousands of CRMs until the loss begins to stabilize. This suggests that most CRMs, among the selected ones, have similar configurations, which is confirmed by the fact that averages calculated over different sets of 10 K random CRMs will be very similar. Early stopping was often needed to prevent overfitting in cell lines with less sources such as CD34, and HeLa to a lesser extent.

**Fig. 5:**
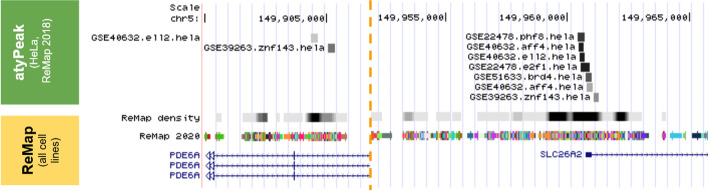
Example of visualisation of atyPeak results in the UCSC genome browser. The results presented here are for the HeLa cell line for ReMap 2018. A darker peak indicates a higher atyPeak score. The annotated BED data files with the corresponding atyPeak scores are available at <https://github.com/qferre/atypeak-files> or as a UCSC browser session at <http://genome-euro.ucsc.edu/s/qferre/atyPeak_hg38>. We can see on this figure an example of rich CRE with many peaks, and a poorer CRE where many correlators for those TRs are missing which predictably has a lower score. As detailed previously, our approach estimates how “typical” each peak is, with respect to the usual combinations between sources (TRs and/or datasets) for a given cell line. As the model is unsupervised, anomaly score thresholds are at the user’s discretion. For example, a large scale analysis might exclude the lowest scoring peaks, but a focused study of a single or selected experimental series may specifically seek low-scoring peaks that might be caused by certain events of interest (mutations, etc.). It is also possible to use high-scoring peaks to detect CREs of interest and use that selection as a filter when looking at other genomic data, like we show here with ReMap 2020

The final score is normalized so that a peak in the average configuration for its source in terms of presence of known correlators will get a score of 750/1000. We provide both normalized and raw scores, at the user’s convenience (Fig. 5).

To confirm the predictions of our model, we perform a comparative analysis with the ReMap 2020 update [26] (Additional file 12: Fig. S7). Presumably, atypical TR fixations as identified by the approach would be less emphasized considering the larger amounts of data processed in this update. We find that the CRMs with the highest update ratio (number of peaks in 2020 divided by 2018) were the ones with lower atypeak scores, suggesting that they were incompletely characterized in 2018 and needed more data. They could also be high-noise regions, although this is unlikely as they were among the richest CRMs in 2018. Supervised data would be required to determine the correct hypothesis. At the individual peak level however, we consider the update ratio for peaks of the same TR within the same CRM, which would confirm the binding of this TR here by drawing from other data. While peaks at any score can have any update ratio as ReMap2020 does not simply replicate all previous experiments a given constant number of times, we find that peaks with a higher update ratio and thus more robust confirmations seldom had a score under 250 in atyPeak.

#### Confirmation of the biological meaningfulness of identified correlations

We also use pairs of Transcriptional Regulators for a second confirmation. These pairs are known to co-occur on the genome, either via their high Jaccard indexes (example in Additional file 13: Fig. S12) or from the literature such as GABPα with ERG in Jurkat [27], ELL2 with AFF4 in HeLa [28], or SFMBT with RCOR1 also in HeLa [29].

**Fig. 6:**
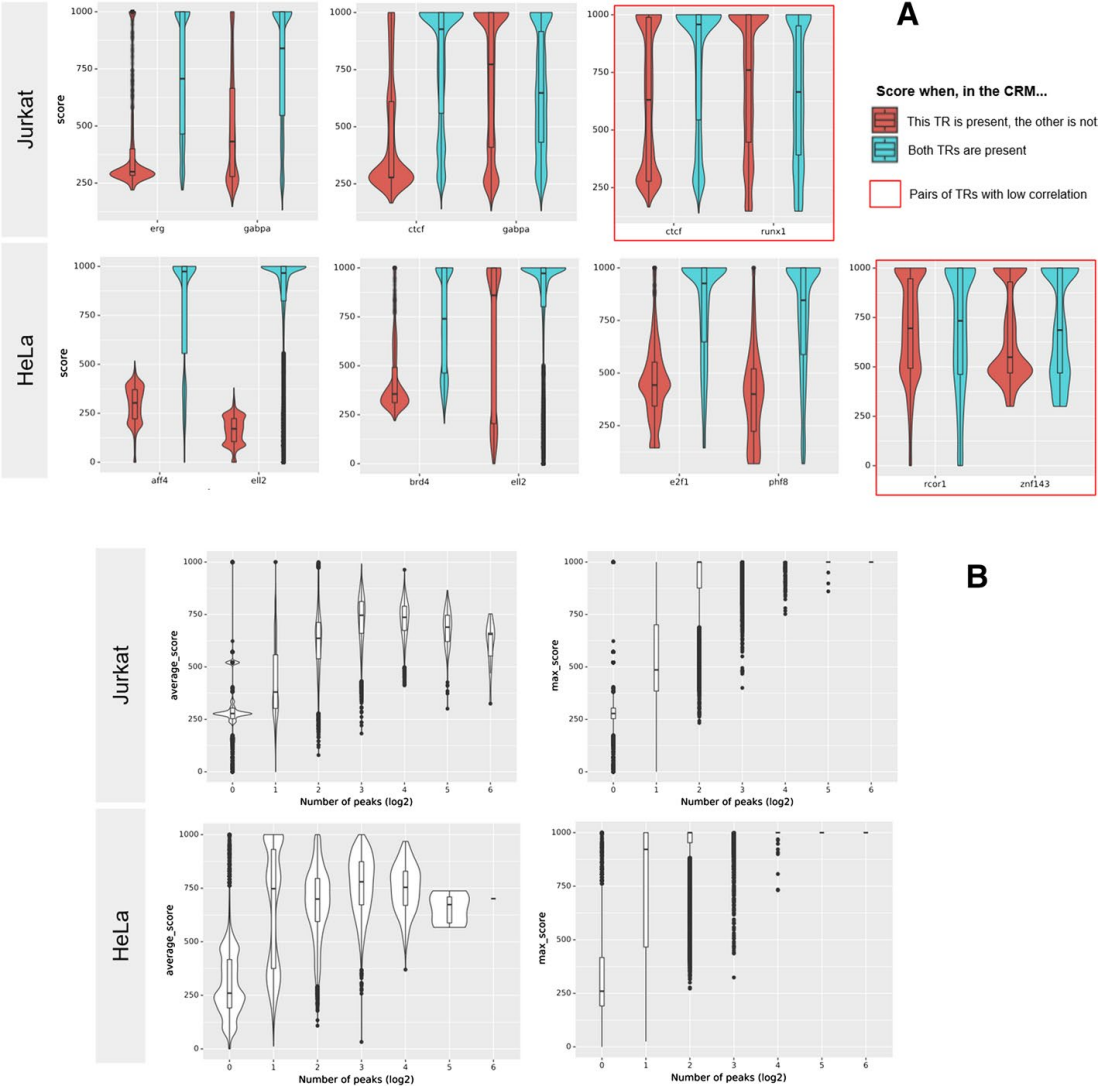
**a** Confirmation of the biological meaningfulness of identified correlations. Scores are considered after applying all normalizations described in Methods. We consider pairs of TFs. For each pair {A,B} we give the score of peaks from A when B is present too in the same CRM (blue) or when B is absent (red). This is the same elementary operation as the Q-score, except we do not average across the X axis but take the actual peak value. Most examples presented are of TRs with high correlation, such as GABPA and ERG in Jurkat which have many common binding sites, ELL2 and AFF4 in Hela, or RCOR1 and SFMBT1 in Hela which are both repressors. When TFs correlate, our model will have learned that and assign higher scores to peaks for a TF when one of its correlators is present. We also provide some counter-examples: CTCF and GABPA in Jurkat have a R coefficient of 0.2 which is high for CTCF but low for GABPA (GABPA is often seen with CTCF, but CTCF has other partners than GABPA) and as such the impact on the score is also unidirectional. Finally the pairs framed in red such as CTCF and RUNX in Jurkat or RCOR1 and ZNF143 in HeLa have a low correlation coefficient. For them, the presence of one TR of the pair has little to no impact on the score of the other. For cases such as AFF4 and ELL2 in HeLa which have one major correlator (namely, each other), the distributions of all scores (blue and red merged) is rather bimodal, as the presence of the other acts as a binary switch. **b** For each processed CRM, average (left) and maximum (right) score of the peaks present in it, depending on the total number of peaks in the CRM. Number of peaks given axis in log2 scale. As Transcriptional Regulators tend to work in complexes, it makes sense that richer CRMs would be on average of better quality. However, the relation is not strictly linear: CRMs with supernumerary peaks likely contain noise, which is reflected here in a lower average score

For each interesting pair A, B of TRs, we consider the distribution of the scores given by our model for A when B is also present in the same CRM, and when it is not. We provide some examples in Fig. 6 with high and poor correlations. We observe that, as we demonstrated earlier, the score given to a peak of a given TR is increased when another TR that correlates with this one is present, and vice-versa for the non-correlating ones. This means that, when properly calibrated, atyPeak learned on its own significant correlations.

Singleton peaks tend to have a lower score compared to peaks found in richer CRMs. This is expected, since CRMs are regions with multiple cooperating TFs, singleton peaks are generally suspicious. This is not simply linear (Fig. 6), which further illustrates that the model learns biologically meaningful correlation groups and not simply that richer CRMs are better.

Some interpretation of the model is possible on real data as well (Additional file 14: Fig. S10). When performing combination mining (Additional file 5: Fig. S11) the learned groups match the expectations about correlating TRs discussed for Fig. 6, such as AFF2 and ELL4 being present in the same group. BRD4 and others are learned together as a more “background” group. SFMBT1 and RCOR1 were learned alone with low phantoms from other sources, although it is justified by their relatively low Jaccard index with the other TRs (Additional file 11: Fig. S8), since as we established overlapping groups are hard to learn. In general, careful interpretation of the learned groups is necessary for cell lines having high frequency imbalances such as Jurkat, or high dimensions such as K562 or MCF7.

## Discussion

We designed an anomaly detection method to identify regulatory peaks that are not part of a cluster of regulatory elements. Our method finds outliers which do not respect the usual sources (TRs and/or experimental series) combinations. Peaks get a higher score when more of their correlators are present, forming a richer cluster. Regions with a high density of high-scoring peaks would be the strongest candidate Cis Regulatory Elements among those sampled (in our study, training was already restricted to a subselection of the densest CREs, so the “best” qualifier will always be relative, as in any unsupervised analysis). This allows for CRE detection taking TR composition into account. Crucially, our unsupervised approach does not require an a priori set of known anomalous experimental peaks, which is seldom available and could bias a model towards the particular kind of anomalies it represents. Our method is an all-in-one approach, meaning that CRE detection, noise detection, etc. are all subsumed under the umbrella of the typicality score.

*atyPeak* learns usual source combinations patterns, while the noise (anomalous peaks as defined in Introduction) is discarded. By focusing on combinations instead of a particular type of anomaly, we de facto indiscriminately correct most of the errors discussed previously. The combinations learned by the model will be based on what is typical in the regions provided in training (for example, our selection contains many gene promoters). We do not fixate on a single type of error, nor do we emit a definitive judgement on peak quality, as it is impossible without supervision. This is made possible by using high-quality ReMap data; indeed, unsupervised anomaly detection presupposes a low proportion of anomalies.

We have validated our approach using artificial data designed to model correlating elements and a noise of atypical peaks. The model autonomously learns multiple *n*-wise correlation groups of sources in both artificial in real biological data. As the underlying task is compression we use comparatively small, simple networks which nevertheless perform well. Hence, our method can be readily used on a laptop from training to application.

### Usage

Our approach estimates how “typical” each peak it with respect to the combinations of sources. Being atypical means the peak is encountered in a different configuration (in terms of presence or absence of correlating sources in its neighborhood on the genome) to those observed in the bulk of examples the model was trained on. In our case, those examples are made of all the CRMs in a single given cell line, since we currently work within one cell line at a time.

As the model is unsupervised, anomaly score thresholds and interpretation are at the user’s discretion depending on their needs. In large scale studies of different and/or redundant experiments, such anomalies are usually noise, especially if the source data was not curated beforehand. As such, a large scale analysis might wish to exclude the lowest scoring peaks in order to denoise, keeping the most common patterns. The threshold depends on the a priori confidence in the data, based on biological reasons (i.e., as much as the assumed False Discovery Rate). However, a focused study of a single or selected experimental series may rely on low-scoring peaks as they might be caused by certain events of interest (mutations, etc.). A low average score for a given CRM also suggests it might be incompletely characterized and missing information about other peaks, instead of noise. We show through a comparison with ReMap 2020 that atyPeak can find, to an extent, peaks were low-confidence and not confirmed by subsequent repeated experiments.

It is also possible to use the UCSC tracks of scored ChIP-seq peaks we provide without using the model itself. An analysis can be restricted to atyPeak peaks with a certain score. Furthermore, when comparing other ChIP-seq data to those tracks, similarity (meaning peaks for the same TFs in the same cell line at the same position) to high scoring peaks suggests this is a typical event. But if the data matches a low scoring peak, or no peak at all, it is atypical. What this means precisely depends on the data being studied, as discussed in the previous paragraphs: it can be noise, or if the user is confident in their data it could be an interesting anomaly worth studying.

While we provide scores for selected ReMap data, our model can be reused to denoise any kind of region database with multi-view sources such as in the ReMap data format (where peaks are independant between cell lines and each have a TR and a source dataset, cf. documentation). Our hierarchical multi-view approach is a type of intermediate fusion, where a first latent space is learned based on one type of combination, followed by learning combinations of it across another dimension. The full tensor represents the CRM; the latent learning by the convolutional kernels is focused on local combinations analogous to CREs (local clusters).

Scaling the information budget is crucial to learn the appropriate correlation groups, and databases with more experiments will require larger models. Furthermore, models with higher budget will be able to remember more patterns, and as such will more rarely give low scores (since fewer patterns will have been discarded). To do so, we propose a Q-score based on whether known correlations influence the rebuilding. We introduce a group-based normalization to correct rebuilding biases and introduce interpretability. We believe these contributions could be applied to other latent variable models, and more generally to any black-box model with arbitrary complex correlations. Both are first steps and warrant further research.

The atyPeak approach has other potential uses. By looking at the added phantoms, it is also possible to interpret the learned correlation groups to find combinations of corroborating experimental series, and regulatory clusters of collaborating TRs. Indeed, we have shown that the presence of biologically known correlators improves scores. The Q-score we propose simply generalizes this observation to all pairs of sources in the data (individual scores for each pair can be extracted); the examples shown in Results were merely handpicked biologically relevant cases.

### Generalization

ChIP-seq protocols, and subsequent quality, can vary wildly. Since our approach learns how experiments are corroborated by others, such differences in quality are self-correcting. Hence, we do not require unified protocols like large consortia (ENCODE) would, and can work with heterogeneous data from multiple provenances. That being said, having more and larger genomic datasets for each Transcriptional Regulator will help.

Other methods may take different perspectives, such as identifying not only shared elements but also those that are specific to specific cell types [30]. In contrast with the latter, atyPeak marking a peak as atypical (low score) only means its local configuration is unusual among the training examples. It does not necessarily mean that its configuration is specific to a given cell line or specific to its source. However, such information may be gained through another potential usage that we find intriguing. It would be imply training atyPeak on one type of genomic region to learn what their typical combinations are, and use this trained model on another type of regions. This would detect local configurations that are different from those observed in the training regions. For example, training on promoters and processing enhancers to identify enhancer-specific correlation patterns; peaks with those patterns will be given a low score. One just needs to be mindful that, like with all ML approaches, sufficient amounts of data must be available for the model to learn from.

While our study focuses on ChIP-seq data, our approach can be generalized to any type of data consisting of a series of peaks, or more generally corroborating time-series intervals from multiple datasets. In genomics, this includes ChIP-exo, ATAC-seq, as planned for future ReMap releases, or even otherwise determined regions like promoters of overexpressed genes in a certain condition. But it could also be used, for example, to compare weather forecasting models. The atyPeak approach could also be applied to many multi-omics problems by changing the meaning of the dimensions, i.e., integrating different assays for different cell lines instead of different datasets for different TRs. More generally, we propose to leverage typical combinations between sources to perform anomaly detection by representing multi-view data as K-dimensional tensors (for K views) and using structures designed to consider those combinations.

To our knowledge, our approach shows the first use of a large-scale meta-analysis of ChIP-seq datasets to corroborate them with each other, using deep learning methods to integrate them in complex combinations. This allowed us to identify and eliminate atypical peaks that do not respect such combinations, resulting in higher-quality data available for genomic analysis.

## Methods

### Materials

#### Data sources

In this study, data is provided by ReMap 2018 [21]. ReMap endeavors to identify and characterize regulatory regions from a large-scale integrative analysis of DNA-binding protein experiments. The 2018 human update uniformly annotated and processed 3180 ChIP-seq experiments, including some biological replicas, creating a catalogue from the analysis of 35.5 million peaks (after merging) for 485 TRs in a variety of cell types and tissues. The regions of interest or Cis-Regulatory Modules (CRM) selected for this study are defined as a region binding at least two different regulatory proteins in all the cell lines and tissues of ReMap, in order to mitigate variation coming from non-standardized sources. A CRM can contains from two to a few thousand peaks, in one (or more) Cis-Regulatory Elements(s). In ReMap, this adds up to 1.6 million CRM; however current estimates point to in the order of magnitude of a few hundred thousands biologically significant ones only.

#### Data selection

We used as a query a subset of the aforementioned CRMs, keeping those with at least 100 peaks across all cell lines for 65,535 CRMs in total, to focus on the densest genomic regions. We processed only a subset of representative cell lines and selected only certain relevant TRs, to reduce the sparsity of the resulting tensor representations. The list of selected sources is present in Additional file 10: Table S1. Our goal was to consider TRs with high biological significance, comparable abundances, and interesting combinations. In practice, for each cell line, we get the TRs with the most experiments, but if a selected TR has a known collaborator further down the line, said collaborator may take the place of a previously selected but isolated TR (e.g., MYC/MAX).

### Autoencoder model

Artificial neural networks (ANNs) are assemblies of neurons, which are logistic units outputting a result dependent on a linear combination of its inputs. In a network, the output of one layer of neurons is fed to the next layer. The weights of each neuron are learned by backpropagation. More specifically, an autoencoder is an ANN whose goal is to learn for each provided example a compressed representation sufficient to rebuild it in the most efficient manner, which entails discarding signal noise [31]. Applications of autoencoders include dimensionality reduction, anomaly detection, information retrieval and image processing.

Here we performed a lossy compression of our CRM representations, i.e., transforming them into shorter vectors capable of returning similar information. When performing a lossy compression, noise and other non-information are the first elements to be lost, but so are fine-grained details. More interestingly, anomalies (in our case atypical peaks) are lost because no regularity involving them is found. Compressing also introduces artifacts, which for us are phantom peaks it was expecting to see.

As any compression algorithm implicitly maps the compressed vector into a feature space, and learning such mappings based on certain criteria (i.e., minimized loss) is a quintessential machine learning task, there is a close connection between machine learning and compression. Deep (convolutional) autoencoders are particularly suited to it [32] and can be tailored efficiently to variations of the problem such as group anomaly detection [33]. The rebuilt image is not a cleaned image, but a compressed one, unlike in denoising autoencoders [34], but those cannot be used here since we have no ground truth, i.e., no a priori information on which peak is good or not.

Existing anomaly detection approaches (see a recent review [35]) solve slightly different problems and ours (anomaly detection based on sources/dimension combinations) is not directly comparable. The closest existing parent is the detection of anomalous vertices in a dynamic graph [36] giving precedent to our approach of giving a score to each vertex at each time step depending on their behavior (see a recent review [37]). Here we use an autoencoder to learn such a score, for which the use of graph convolution is precedented [38] although instead of edges we have multi-view bags of items. Another related approach is multivariate time series anomaly detection, but here we seek to label anomalous features, not anomalous points.

#### Data representation

Each putative CRM is represented as a 3D tensor $T \in {\mathbb{R}}^{3}$. This tensor of peak presence contains a representation of ChIP-seq peaks falling into this region: the *x*, *y*, *z* dimensions are respectively the nucleotide position, the experiment/series ID, and the Transcription Factor involved in the ChIP-seq. Each cell line is analysed separately. The value at each position of the tensor is 1 if there is a peak, 0 otherwise. CRM longer than 3200 bp are truncated, and those shorter are padded with zeros (3.2kbp was the 9th decile of length). The use of an additional third dimension for parallel integration of different data views is precedented, even in bioinformatics, and there is precedent for the use of tensor decompositions on such representations [39]. Our auto-encoder approach inscribes itself in this lineage.

We then downscale the tensor by a factor 10 along the X axis (“squishing”) since the data has low granularity along that axis to allow the use of smaller, easier to train convolutional kernels. Also, to counteract lower rebuilt values at the margins of the tensor in CNNs (Convolutional Neural Networks), we add a padding of meaningless zeroes at the beginning and end of the X axis instead. Its length is twice the convolutional kernels’ length on each side.

However, CNNs have trouble training on sparse data. Coincidentally, encoder-decoders handle sparse data better when combined with a sparse training strategy [40] where the density of tensors used in the training is tweaked during said training. This inspired our crumbing approach: in real biological data only, to help the model learn in spite of the sparsity, we also add crumbing (Additional file 8: Fig. S9) where for each tensor element where there is a nonzero value of *v*, we add 0.1*v in each position in the same Y or Z axis as a hint. Crumbing is cumulative.

#### Model architecture

The structure of the model is detailed in Fig. 1b. Our model has two parts: an encoder creating a latent representation and a decoder retrieving the original tensor. As with all autoencoders, the model is trained to try to rebuild the original CRM representation as its output. The full model parameters are available on GitHub. Our model was implemented using Keras 2.3 [41], with Tensorflow 1.15 and NumPy 1.18.1 [42].

#### Convolutional encoding

The CRM representations are viewed through sliding convolutional filters, to focus on correlations between the TRs and datasets. A convolutional filter gets as input a slice of a matrix and outputs a weighted sum of its elements, with the weights forming the filter proper. The first layers are two successive convolutions with two different types of kernels (combinations of datasets, then combinations of TRs).

Let *n* be the number of TRs in the cell line, *m* the number of datasets and *k* the size of the convolutional kernels. As a result, the kernel shapes are (*k*, *m*, *l*, *channel* = 1) for the first, and (*k*, *l*, *n*, *F*) in the second where *F* is the number of filters in the previous layer. As there is no ordering to the datasets or TR, we perform a depthwise convolution and read the entire dimension at once. Default *k* is 20. We use only one layer per dimension. We use few kernels, lower than the later Dense layer size, creating a bottleneck [43], but larger Dense layers still improves rebuilding (Fig. 3).

The combinations are learned over a short window across the region given by the variety and stride of the convolutional kernels. Convolution filters have a kernel regularisation of 2.5E−3 by default and Dense layers (see below) have low Dropout regularisation (10%), except for the encoded layer which has none so it can specialize. We observed that a stacked approach of one dimension at a time can lessen training problems associated with large number of dimensions.

Convolutional filters are known to be useful in finding combinations of elements across dimensions, including in biological sequences [44]. Multi-view integration is traditionally done by using different strides for the filters, or by processing each view followed by a feature fusion [23]. In contrast, as our dimensions are incomparable, we express a hierarchy between our two dimensions by integrating datasets combinations first, learning a first latent space which is then passed to another convolutional layer, which learned its own latent space based on TR combinations (across another dimension) of the values of the first latent space.

#### Integrative layers and decoding

The convolutional layers are followed by 4 regular (Dense) integrative layers, to learn complex combinations. On each layer, only the last dimension (filters) provides weights, resulting in Time-Distributed layers with no communication along the X dimension. The ReLu activation function is used.

We obtain an encoded dimension in the fourth Dense layer. For the decoding, we consider each element of the non-human-readable encoded dimension as a latent variable. A first layer of convolutional filters reads the entire dimension once to produce a first decoding, and a second and final layer has one filter per source (TR-dataset pair). This is done because unlike classical images, there is no order to the features. The final layers perform a reshaping of the result back to the original tensor shape.

There is no communication along the X axis, unlike NLP models such as Transformer or LSTMs, as we focus on local combinations. However, along the other axis of the encoded dimension, the encoding layer has access to the state of all other learned neurons making this partly reminiscent of a transposed attention mechanism [45]. Custom time-based layers and constraints could be added here. This is not necessary to work with large, overlapping ChIP-seq peaks, but might be needed to integrate the ReMap 2020 new ChIP-exo and DAB-seq data.

As such, even though the full tensor represents the CRM; the latent learning is focused on local combinations analogous to CREs (local clusters) in a window the size of the convolutional kernels.

#### Loss used

The loss used when training the model is the Mean Squared Error, or L2 loss. Hence, when the model adds phantoms from the same correlation group, it must lower the value of the original peaks. This forces compromise compared to a L1 loss, since $$\left\| {\left( {\begin{array}{*{20}c} 0 \\ 2 \\ \end{array} } \right) - \left( {\begin{array}{*{20}c} 1 \\ 1 \\ \end{array} } \right)} \right\|_{2}^{2} < \left\| {\left( {\begin{array}{*{20}c} 0 \\ 2 \\ \end{array} } \right) - \left( {\begin{array}{*{20}c} 2 \\ 0 \\ \end{array} } \right)} \right\|_{2}^{2}$$. Using L2 loss on an autoencoder has been previously shown to be effective to perform denoising even when no cleaned images, only corrupted/noisy versions, are available [46].

The best results were obtained with the Adam (“Adaptive learning rate for sparse data”) optimizer [47], which is effective on problems featuring large data and very noisy and/or sparse gradients. It adapts the learning rate based on gradient moments. Our base Learning Rate for the Adam optimizer is 1E−4, which is 0.1× its default. We keep the model with the lowest loss of all the epochs during the training process. Training is stopped when losses stops diminishing with a patience of 5 and a minimum delta of 2.5E−4 (chosen empirically) to consider it to have diminished significantly. Batch size is 48 CRMs with 10 batches per epoch in artificial, 48 in real data. We can also use a weighted loss by apply weighting to the loss for each dataset or TR separately.

#### Anomaly score

Finally, each peak gets an anomaly score based on the autoencoder reconstruction error. The better the peak according to the model, the higher the score. Such an approach has precedent in signal processing [48].

We define an anomaly score to compare a tensor *X* to its rebuilding by a model noted *h*(*X*). We have *A* the anomaly score tensor defined as:

$$A\left[ {x,y,z} \right] = \left\{ {\begin{array}{*{20}l} 0 \hfill & {{\text{if}}\;X[z,y,z] = 0} \hfill \\ {1 - \frac{\begin{gathered} \hfill \\ X[x,y,z] - h\left( X \right)[x,y,z] \hfill \\ \end{gathered} }{X[x,y,z]}} \hfill & {{\text{else}}.} \hfill \\ \end{array} } \right.$$

Dividing by the original score accounts for the potential crumbing. The score of each peak is the maximum value in *A* across all nucleotides of the peak. This is necessary to correct cases where near vicinity peaks will get high score only on the parts where they overlap and because peaks smaller than the convolutional filter’s length will get a lower rebuilt score as a result. Peaks can sometimes be divided between two (or more) of our 3200-bp windows, getting one score for each rebuilt matrix: we merge them by giving them a score that is the mean of each.

#### Normalization of correlation group biases

This normalization aims to correct bias in rebuilding based on the learned correlation groups. We calculate a weight for each source (meaning each {dataset, TR} pair), based on the following steps, to be applied to all anomaly scores computed for this source.

*μ*_*X*_(*T*) designates the 2D matrix of the mean across the X axis (region size) of the 3D tensor *T*, and *max*_*X*_(*T*) its maximum. *M*[*s*] designates the value of the matrix M for the current source. *h*(*T*) is the tensor obtained as output when passing as input the *T* tensor to a trained atyPeak model. We note *F*_*t*_ a “full CRM” which is a 3D tensor representation where all possible sources with abundance higher than zero are present along its length with a value of 1. Let *F* = *μ*_*X*_(*F*_*t*_). The correlation group of a source can be estimated (see Interpretability) by preparing a CRM containing only a peak for the given source along its entire length denoted *U*_*s*_. We get such a request mask as *R* = *μ*_*X*_(*h*(*U*_*s*_)).

The first step corrects for intra-group biais in rebuilding, due to learning bias (usually too high learning rate) or abundance differences within the group. There, the sum of the rebuilt CRM will be biased too. We get the first weight $k_{1} = {{\sum {h\left( T \right)} } \mathord{\left/ {\vphantom {{\sum {h\left( T \right)} } {\sum T }}} \right. \kern-\nulldelimiterspace} {\sum T }}.$

For inter-group bias, recall that the rebuilt value of a peak (value in *h*(*T*)) is proportional to how complete its correlation group is. But group have different sizes, cf background group and different groups. Our goal is that peaks gets the same score where their group’s current completeness relative to its average completeness is the same. We define occupancy for a CRM for the current source as M*max_*X*_(*U*_*s*_)*IW where *M* = *R*/*max*(*R*) and *IW* is the matrix of intra group weights for all sources. These are pointwise multiplication, not matrix products. We use a Monte Carlo approach by iterating over a portion of all CRMs and calculating the mean of all their occupancies *μ*(*θ*), excluding zeroes to get only the correlators when the source is actually present, and self-correct for relative loneliness. The final second weight *k*_2_ = *θ*_*F*_/*μ*(*θ*) where *θ*_*F*_ is the occupancy calculated of *F*_*t*_.

Thirdly, if a source is in several groups, phantoms from several groups can accumulate and will not be seen at step 2. We evaluate how much the sources that are not in the request will contribute. We calculate the mean and max negative occupancies (*η*) exactly as above, except we use a negative mask *M*_*n*_ instead of request *R*, where *M*_*n*_ = (*F* − *R*/*R*[*s*])*F[*s*]. We ponder by the average presence of these other peaks to get *k*_3_ = 1 − ((*h*(*F*)[*s*]/*F*[*s*])*(*μ*(*η*)/*η*_*F*_)).

The final weight is *k* = *k*_1_ * *k*_2_ * *k*_3_. To prevent overcorrection of sources that were not learned by the model, all *k* are capped at 10. For now, having a CRE with more peaks than average results in higher rebuilt values, as we consider that for CREs in particular more TRs mark denser/better CREs. This assumption could be changed here by penalizing values above the corrected average quality.

The final step consists of centering and reducing/normalizing the scores by TR, under the assumption that no TR is inherently of a better quality than the others. Having more correlators (i.e., data less sparse for the same dimension, more datasets per TRs) is a benefit. For each peak, if their score at this step is *s* their final score is $s_{f} = 750*\left( {1 + \frac{{s - \mu_{TR} }}{{2\sigma_{TR} }}} \right)$, where *μ*_*TR*_ and *σ*_*TR*_ are respectively the mean and standard deviation of scores observed for this source’s TR at the previous step. Note that scores are usually not normally distributed.

We center around 750 to use a larger part of the score scale for cases where the local cluster (CRE) is less complete than average, which are the cases we want to mark. If you choose to use a non-normalized score, compare each score to the median score for its source. This normalization is a step in the right direction that independently moves score averages for different TRs closer (Additional file 15: Fig. S13) but warrants further research.

### Training and interpreting the model

We provide scripts to directly process a BED file in ReMap format with diagnostic figures and usage instructions in the Readme.

#### Impact of data characteristics/scaling on required information budget

As we discussed in Results, the information budget determines the aggressivity of the compression. It depends on the relative information budget. It is the ratio between the quantity of information to be learned in the data (itself a function of the number of TR and/or dataset combinations) and the model’s entropic capacity (how much information can be stored in the compressed representation). Adequate hyperparameter tuning is a widespread problem in deep learning as a higher information budget will predictably increase the model’s Vapnik–Chervonenkis dimension and make it more prone to overfitting.

In our case, the entropic capacity is mostly increased by increasing the dimension of all Dense layers and the number of convolutional filters on one side (more is higher). But also by diminishing the learning rate (LR) on the other which was often necessary to reach lower losses, even with all other parameters constant. We saw in Fig. 3 that to achieve the same aggressivity, the required entropic capacity scales up with the quantity of information in the data. Figure 2B, on the contrary, is an example of overprecision. Larger dimensions (more datasets and/or TRs in the database) require a higher information budget, even with no additional information (Additional file 3: Fig. S3B). However, Lower Learning Rates are more of a necessary condition than larger models to reach higher precisions with higher dimensions (Additional file 3: Fig. S3C). Learning larger correlation groups (composed of more sources) is also harder.

The most frequent sources are learned in more precise groups, while the rarer ones appear often grouped together in more “background” groups. All groups, but especially the latter, are not expected to be fully complete (meaning all the sources are present) in the real data. More generally, sources that are comparatively too rate (empirically $${\raise0.5ex\hbox{$\scriptstyle 1$} \kern-0.1em/\kern-0.15em \lower0.25ex\hbox{$\scriptstyle 5$}}$$ difference) may be completely disregarded by the model as they are seen as systematic noise. All those tendencies are more visible in high-dimensionality examples or those with higher imbalances, and can be alleviated by using a weighted loss: Additional file 4: Fig. S4A shows that dimensions with a higher weight will be focused on and get more precise groups.

We also show in Additional file 4: Fig. S4B that the model is capable of learning overlapping groups (where the groups are “G1” and “G1 + G2” instead of “G1” and “G2” like in Additional file 1: Fig. S1) However, it required learning adjustments with higher weighting on the rarest dimensions to direct the learning, and more importantly early stopping. With a variety of other parameters, peaks in G2 produce only marginal phantoms for G1, or we get too precise or non-homogeneous groups (Additional file 4: Fig. S4C). Note that G1 will often not produce phantoms of G2 (although it should and does sometimes happen, like in HeLa) so be careful to look at the estimated groups for all sources when interpreting the model. Relatedly, even in non-overlapping groups the watermark (i.e., the lonely control peak we added at the same position to most of the CRM that does not particularly correlate with other groups, see Artificial data) does not create phantoms anywhere else. However, watermark phantoms are produced by peaks from (certain sources in) the G1 and G2 groups. The rarer of those two groups often erroneously produces stronger phantoms, a tendency reduced when this rarer group is weighted more.

#### Loss and training

Due to the high dimensionality and sparsity of our data, we used lower Learning Rates (Additional file 9: Table S2) and large batches to counter overfitting and batch effects. We also used early stopping in most cases, in most cases stopping even before a loss low plateau is reached to prevent the model from adding bias in a futile attempt to improve.

With different random seeds, we observed over several runs small but real deviations in scores and estimated correlation groups. As with most machine learning approaches, we recommend averaging over several runs (2–3) for both these applications.

Training the model takes around 10–30 min per cell line for smaller models (HeLa) and 1–5 h for larger ones (K562 and MCF7). However, reading and processing the source BED files is a large part of this time and the approach is not CPU bound. Times given on an i7-7820HQ and on an SSD drive. GPU use did not significantly improve running times. Production of the resulting BED file after training is also time consuming (around 12 h for K562 but 40 min for HeLa), so it is advised to check some rebuilt matrices before proceeding.

#### Interpretability

To interpret the latent variables in the encoded dimension (Fig. 1, Additional file 6: Fig. S6 and Additional file 14: Fig. S10), we use a gradient ascent method to build an hypothetical CRM tensor that would maximally activate each individual row in the encoded dimension layer [49].

We seek $TM = \left\{ {\forall i \in \left[ {1;\# \left( E \right)} \right],\mathop {{\text{argmax}}}\limits_{T} a_{E,i} (T)} \right\}$. We add some blur at regular intervals on the Y and Z axis during gradient ascent for more natural looking results. By default we use a learning rate of 1, 50 steps in gradient ascent, and the blur standard deviation is (*σ*_*x*_, *σ*_*y*_, *σ*_*z*_) = (0.2, 1*E* − 2, 1*E* − 2) applied every 5 steps. For each latent variable of the encoded dimension the gradient is calculated across the entire length. As the Dense layers are not connected across the X axis, we are considering local combinations. Since this is not the next-to-last layer, the final result will be a complex non-linear combination of those variables. This should instead be seen as a highlight of the model’s focus during learning.

Another type of interpretability is based on the same procedure used in the normalization (Additional file 5: Fig. S11). We create a CRM representation *U*_*s*_ that is empty except for one peak for a given source along all its length. By looking at *R* = *h*(*U*_*s*_), we see what phantoms are added by the model, and deduce these are part of the same correlation group as the source we are considering. Due to the peculiarities mentioned above when learning overlapping groups, look at all the sources’ estimated groups, as a source A may impact the score of B without B appearing in A’s estimated group. Passing *R* does not always result in values of 1 due to complex nonlinearity, but it is a good approximation.

Note that a learned correlation group of “ABCDE” does not necessarily mean ABCDE are always found together, as seen in artificial data where the model learned the entire G1 and G2 group, which almost never found complete in the artificial CRMs. As such, rarer sources can be grouped in more background groups without necessarily being a complex. For both interpretabilities, negative weights are likely due to sum averaging and should not be focused on. Indeed, the rebuilt tensor is not simply the sum of the estimated correlation groups for the sources present.

### Q-score quantifies the quality of the reconstruction

Autoencoders are usually evaluated based on the reconstruction error [50]. Here, we instead propose a new method more adapted to the problematic. To rigorously choose the information budget, we propose to verify that the model correctly learns generated pairwise correlations. On one hand, if two dimensions (datasets or TF) correlate, finding them both together in the region of interest should result in a higher score for them than when they are found alone; on the other hand if they do not correlate, this should have no impact. To estimate this we design a Q-score, which is lower in better models.

The Q-score is defined as:

$$\forall \left( {i,j} \right) \in \Lambda ,\;Q = \sum\limits_{i,j} {\sqrt {A_{i} *A_{j} } *\left( {P + R} \right)}$$

where

$$C = \left[ {R\left( {i,j} \right) > \hat{\mu }\left( R \right)} \right]$$

$$\epsilon = 0.05*\# \left( \Lambda \right)$$

$$P = \left( {C - \left[ {P\left( {\mu \left( {alone} \right) = \mu \left( {both} \right)} \right) < \epsilon } \right]} \right)^{2}$$

$$R = \left( {C - \left[ {P\left( {\mu \left( {phantom} \right) = \mu \left( {none} \right)} \right) < \epsilon } \right]} \right)^{2}$$

Here Λ is a set of all TRs and all datasets (so all possible Y and Z dimensions, excluding the X dimensions of peak position) and the brackets are Iverson brackets denoting indicator variables. Note that we only compare TRs with other TRs and datasets with other datasets, because a dataset and a TR are not mutually exclusive and issues can arise when considering a dimension that is only present as noise when another is present. For the same reason, we consider only positive correlation coefficients later.

*C* asserts whether the Pearson correlation coefficient between the two considered dimensions is higher than the mean correlation coefficient. It is calculated on the tensor representations of the CRMs at the nucleotide level.

For *P* and *R*, we take 10thousand CRM tensor representations *T* and their rebuilding *h*(*T*). For each of them, we record the values for the (*i*, *j*) dimensions of interest (averaged across X axis). We compare the average rebuilt value of A in different scenarios: For *P*, when a peak of *i* was present in *T*, does presence of *j* in the same CRM result in a higher rebuilt value for *i*? And for *R*, when *i* was absent, does the presence of *j* result in higher phantom values than when *j* is absent? To perform these comparisons, we use a Welch test to determine whether the means are different. We use a Bonferroni correction by using a *p *value of 0.05/#(Λ). We then weight the result by the relative abundance of the dimensions *A*_*i*_ and *A*_*j*_. We do not normalize the scores with the procedure discussed before because we compare a source with its own values.

### Artificial data

We use artificial regions to confirm the model can discover correlation groups. They are meant to approximate real genomic CRMs, hence the generation process and parameters are based on true data.

We define a probabilistic model to generate the artificial data. The output of this model is an ensemble *P* of peaks, whose characteristics are: their start and end, the IDs of the TF they represent and the experiment they belong to. Hence we have $P = \left\{ {\left( {s_{i} ,e_{i} } \right),act_{i} \in \left\{ {0,1} \right\},TF \in N,series \in N} \right\}$ which is then converted into a 3D tensor representation, as explained in Data representation. The generation itself consists of three steps detailed below. Each step is run once per generated artificial CRM. Unless specified otherwise, all random variables used are Poisson R.V. of *λ* = 1. See Additional file 1: Fig. S1 for an illustration of the dimensions.

First, we place a control peak called a watermark along all the length of the CRM for the 1st TF in the 1st dataset, representing ubiquitous TRs. It will be very frequent but not particularly correlated with other sources and so form its own correlation group. It has a customizable probability (default 75%) of appearing, to prevent the model from learning it and only it when it is too frequent.

Second, we want to place a stack of correlating peaks from different TRs and datasets, at roughly the same positions. The stack will belong to one of two or more TR “correlation groups”. The groups are made by splitting the set of all in TRs in two, or more, or by making groups of 4. Group choosing probabilities are equal by default but can be weighted.

Only one such group is picked per generated artificial CRM. We then pick a common center for the peaks, uniformly randomly across the region. Now, we pick *K* + 1 datasets without replacement among all predetermined reliable datasets (by default, the last half of them). In these datasets we will place *N* + 1 peaks. For each peak to be added, we randomly select *P* + 1 TRs from the current correlation group with replacement. *N*, *K* and *P* are random variables. For each TR selected, separately move the center by a distance *j*_*d*_ (uniform R.V. between -200 and + 200), take a peak length randomly of *L* (*L* is a log-normal RV of *μ* = 250, *σ* = 0.25) and finally, write the exact same peak among all the datasets selected previously. Note that since artificial data draws peaks at random, there is a larger number of possible combinations than there is usually in real data of the same dimensionality.

Third, noise peaks are placed uniformly randomly from all datasets and TRs to represent false positives and atypical peaks, which by nature do not respect existing correlation groups. To represent false negatives, each peak has a probability *t* = 1/4 of being removed at this tep. Then, we randomly position *F* + 1 peaks (*F* is a R.V.) by drawing randomly their characteristics like previously. Noise cannot be placed in the watermark.

## Supplementary Information

**Additional file 1: Fig. S1.** Systematisation of artificial data analysis in 10 thousand CRM. For both **a** and **b **we use a model with deep dimension of 32, 16 convolutional filters, and LR of 1E−3. We compare a situation where such a model is too precise in **b** where the artificial data dimensions are 6 × 4“ with a situation in **a **where such a model is adequate.The left plot gives the distribution of rebuilt (max across) values of peaks depending on their type: respectively noise in reliable (R) datasets, noise in unreliable (UR) datasets, phantoms (peaks added that were not present in original matrix) and stack (peaks that were in the stack of added peaks). See Additional file 2: Fig. S2 for details. The color gives the correlation group of the peak (belonged to the same group as the group where the stack of peaks was placed for this CRM, or different). “Brothers” is the total number of peaks in same line or same column (summed). In both cases, the stack of peaks (and watermark) are correctly rebuilt, and phantoms of a high value are added in the same correlation group as the stack, but not in the other group. The correct rebuilding of the watermark shows lonely peaks can still be learned when frequent. When noise is added in the (R) datasets, it will not be part of a stack hence its usual correlators will not have been added : lacking its correlators, it atypical by our definition and gets a lower value due to this, not just because it is lonely. Noise in (UR) is discarded by the model due to its rarity. Noise scores are higher in (b) as the groups have less members, and a single noisy source represents a larger proportion of the total group learned than in (a)

**Additional file 2: Fig. S2.** Example of an artificially generated region representation and relevant groups, in 8 × 8 dimension. The small colored lines are only visual aids. The X axis (bottom left) is the position along the region, the Y axis (bottom right) is the dataset number and the Z axis (right) is the Transcriptional Regulator number. Datasets are split in half between “Reliable” (R) which will contain both the stack of true peaks and noise, and “Unreliable” (UR) which will contain only noise. TFs are split in the G1 and G2 groups. They can optionally be split in more than two groups. We first place a stack of peaks around a common position. These peaks will belong to either the G1 or G2 group. As a result, sources within the G1 or G2 group will correlate with each other, but will not significantly correlate with sources from outside their group. We then add noise uniformly randomly that can belong to any dataset and TR, representing anomalies. Finally a control watermark peak is added with, usually, 75% probability, representing ubiquitous TRs. All values in the tensor are 1, denoting presence. More details are available in Methods

**Additional file 3: Fig. S3.** Learning biases and budgets. **a** Using artificial data with group selection odds for G1 and G2 set at $${\raise0.5ex\hbox{$\scriptstyle 2$} \kern-0.1em/\kern-0.15em \lower0.25ex\hbox{$\scriptstyle 3$}}$$and $${\raise0.5ex\hbox{$\scriptstyle 1$} \kern-0.1em/\kern-0.15em \lower0.25ex\hbox{$\scriptstyle 3$}}$$ instead of equal. The values given in rebuilding are still only dependant on group completeness, the difference in abundance between the two groups does not influence the result. The model had 16 filters, deep dimension of 32 and LR of 1E−4. **b **Using two equiprobable non overlapping groups, as per Additional file 2: Fig. S2. The only difference between “padding” and “No padding” is that a padding of 12 lines (TFs) of zeroes were added to the matrices passed to the model. The model had 96 filters, 256 deep dimension, LR of 1E−4. This shows that even where there is no new information (in the left, the two groups G1 and G2 are still in the two top-rightmost 4x4 blocks), the precision is lower for the same model when the data dimensions are larger. **c** Model trained with artificial data, with 4 correlation groups, 64 filters and 600 deep dimension. The correlation groups predefined at data generation are reminded by the dotted lines. In spite of the very large information budget of the model, a LR of 1E−4 was not enough to reach an over-precise learning. Overprecision was achieved only with a much lower LR of 1E−5, which demonstrates that to reach increased precision (less aggressive compressions) lower LRs are more of a necessary condition than large deep dimensions

**Additional file 4: Fig. S4.a** Usage of a weighted loss. We use a model with 24 filters, deep dimension of 64 and a LR of 1E−4. We use artificial data with the same generation process as usual, as detailed in Additional file 2: Fig. S2. However, we assign a weight of 10 in the loss to all UR datasets (0–4 included), which means that when computing the loss errors on these dimensions count 10x as much. Similar models without weighting would entirely discard peaks from suich UR datasets (Fig. 2), but now they are now learned with high precision, almost individually, while the precision is not as great for the non-weighted sources. This highlights the role of weighting in directing the learning towards specific sources and on the process of learning in general. **b** and **c** Overlapping groups. For this figure, when generating the data the two possible groups to choose from when placing the stack were not “G1” and “G2” but “G1” and “G1+G2 = all TRs”. This means the second groups overlaps with the first, and in fact contains all its sources plus its own exclusives. Both groups had 50% odds of being selected. Such overlapping groups are hard to learn and needs careful parametrization, as we explain in Methods. This requires use of 2× weighting for the sources of G2, and early stopping at 16 epochs. In B, the overlapping groups are learned properly and we see that G2 produces phantoms for G1. The model used had 16 filters and 32 deep dimension and a LR of 1E−4. **c** Is an example of difficulties that can be encountered. Done with a model of 24 filters, 64 deep dimension, a higher LR of 1E−3 and crucially, no weighting. The sources of G2 still produce some phantoms for the sources in G1, but those are much fainter, and the rebuilt groups are not homogeneous. Note that a lower LR of 1E−4 for this LR resulted in increased precision as would be expected, with more precise groups for G1 and no overlapping phantoms

**Additional file 5: Fig. S11.** Estimating the correlation groups certain sources belong to. This is done in the HeLa cell line, with the legend indicating respectively the Transcriptional Regulator and dataset concerned. As detailed in Methods, for each given source we create an empty CRM representation with a peak along its length for this source only, and pass it to the trained model. The result, shown above, is the sum across the X axis of the rebuilt tensor. The cross ‘+’ pattern is due to crumbing, one needs to be mindful of it when interpreting. Note that several sources (BRD4, SFMBT1) are present in more than one group

**Additional file 6: Fig. S6.** Example of interpretability in artificial data. **a** The bottom figure presents an example of artificial tensor on the bottom, and the top heatmap gives the squared activation of encoded dimension when this representation is passed to the model. The parameters used are the same as Fig. 2a. **b** Gives several ur-examples (average across X axis) of CRMs that would maximally activate one row in the encoded dimension (rows in top left). The focus is mostly on the correlation groups as a whole. This is useful as a focus map to see where the model focuses its learning, but the final rebuilt tensor is not simply a linear combination of those, as evidenced by the fact that some ur-examples focus on both correlation groups. Some dimensions are never used, and redundancies were observed. Note that watermark is visible in those ur-examples only when it is not added 100% of the time (and therefore is a variable and not a constant)

**Additional file 7: Fig. S5.** Q-score matrices giving the contributions for each pairs of dimensions for two models from Fig. 3. Lower is better. **a** Corresponds to the model with 8 Deep dimension and **b** to the model with 256 deep dimensions. The numbers of both the X and Y axis have the same significance : 0–7 represent datasets 0 to 7, and 8–23 represent the TRs 0 to 15. The Q-score assesses, for each couple of dimensions (datasets with datasets and TRs with TRs) if the presence of one results in a higher score when present for the other, or in higher phantoms. The better model has lower Q-score, as the 8 groups were learned properly. The Q-score is currently a work in progress but is informative as to the larger trends to learning. More details are presented in Methods

**Additional file 8: Fig. S9.** Crumbing. On the figure, values from low to high and red to blue. Thin lines are a visual aid. Crumbing is added to real data matrices to fight sparsity. For each nonzero value in the original CRM representation at position [x,y,z], 1/10th of this value is added to all positions at [x,:,z] and [x,y,:], meaning for all datasets sharing the same TR and all TRs sharing the same dataset, forming a “+” pattern. This is necessary because on very sparse data, such as the real data tends to be, the model can easily fall in the learning trap of rebuilding a completely empty tensor

**Additional file 9: Table S2.** Parameters used when processing real ReMap data. This provides a baseline based on the dimensions of the data for our cell lines. Dimensions provided are for comparison purposes, to provide a baseline. Other parameters (regularisation, etc.) have the same value for all cell lines, given in the Methods section in the paper. The nonzero sources gives the number of sources encountered often enough (at least one in several thousand CRMs depending on cell line) that a normalization coefficient was computed for them

**Additional file 10: Table S1.** List of datasets and Transcriptional Regulators from ReMap 2018 used in this study. Datasets IDs are grouped by prefix in the table: for example, “GSE52924” and “GSE54344” are grouped as “GSE: 52924, 54344”. For the cell lines, if variants are present in the ReMap data, they are not kept. For example, CD34 does not include CD34_condition1

**Additional file 11: Fig. S8.** Demonstration of overlapping groups learning by the model. **a** Gives a true HeLa CRM and its rebuilding, and **b** is the same CRM after removing all peaks excepted for those belonging AFF4 and ELL2. Even though they are a group almost by themselves (Additional file 5: Fig. S11), removing all other peaks results in AFF4 and ELL2 having a lower score in the rebuilding of A than B as BRD4 and others also contribute to their group, even though they are learned in another group. This confirms overlapping groups are possible in real data, but subject to caveats describes in Methods. Figures give the maximum across the X axis. Parameters are given in Additional file 9: Table S2.

**Additional file 12: Fig. S7.** Comparison between ReMap 2018, ReMap 2020 and predictions made by atyPeak. **a** For each CRM (random subselection of 10,000), number of peaks in either update and mean atyPeak score in 2018. Low scoring CRMs tend to have less peaks, and have proportionally more peaks added in 2020. **b** For each peak (random subselection of 5000), number of peaks in 2020 for the same TF in the same CRM divided by the number in 2018, potentially from any number of databases. Restricted to peaks in CRM with average score of at least 500 to prevent bias described in subfigure A. Log scale is used for the score to emphasize the low-scoring peaks. We see that peaks with a score of under 250 are more rarely confirmed in 2020

**Additional file 13: Fig. S12.** Jaccard index A∩B/A∪B for HeLa Transcriptional Regulators. Based on the CRM representations we created for this data. Those are provided for comparison purpose and interpretation of the extracted correlations learned by the model in HeLa

**Additional file 14: Fig. S10.** Analysis of a trained HeLa model. **a** Presents some ur-examples (maximally activating CRMs for each element of the encoded dimension) summed across the X axis, calculated on a trained HeLa model. **b** Is HeLa correlation matrix between all dimensions like in the Q-score, **c**is the Q-score contributions. We observed that using a higher deep dimension can still help reach a lower loss, even with redundancies in the ur-examples, including on real data

**Additional file 15: Fig. S13.** Mean score per TR in HeLa before and after applying the normalization detailed in “Normalization of correlation group biases” in Methods, but before centering and reducing it based on the mean score for each TR. In summary, this normalization corrects biases due to average completeness differences between groups. After applying this part of our normalization, the means tend to be closer between the different TRs, correcting the various biases we detailed. For example, sources learned as part of larger groups like BRD4 (see groups in Addiotional file 5: Fig. S11) get a needed boost to their score

## Data Availability

The datasets analyzed during the current study are based on ReMap 2018 and are available in our GitHub repository at <https://github.com/qferre/atypeak>, along with the model source code. Treated data files with scores for the considered ReMap peaks are available as a UCSC session at <http://genome-euro.ucsc.edu/s/qferre/atyPeak_hg38> (Fig. 5), or as BED files with supplementary diagnostic data at <https://github.com/qferre/atypeak-files> and on the ReMap website at <http://remap2020.univ-amu.fr/download_page>.

## Abbreviations

TR: transcriptional regulator
CRE: cis regulatory element
NGS: next generation sequencing
TF: transcription factor
CRM: cis regulatory modules
TFBS: transcription factor binding site
TRBS: transcriptional regulator binding site
FDR: false discovery rate
IDR: irreproducible discovery rate
DNN: deep neural networks
PCA: principal component analysis
NN: neural network
LR: learning rate
ANN: artificial neural network
CNN: convolutional neural network
BED: browser extensible data
NLP: natural language processing
Q-score: quality score
