# Supplementary figures and images for: Anomaly detection in genomic catalogues using unsupervised multi-view autoencoders
Source: BMC Bioinformatics. 2021 Sep 25;22:460. doi: 10.1186/s12859-021-04359-2 (PMC8467021; doi:10.1186/s12859-021-04359-2)

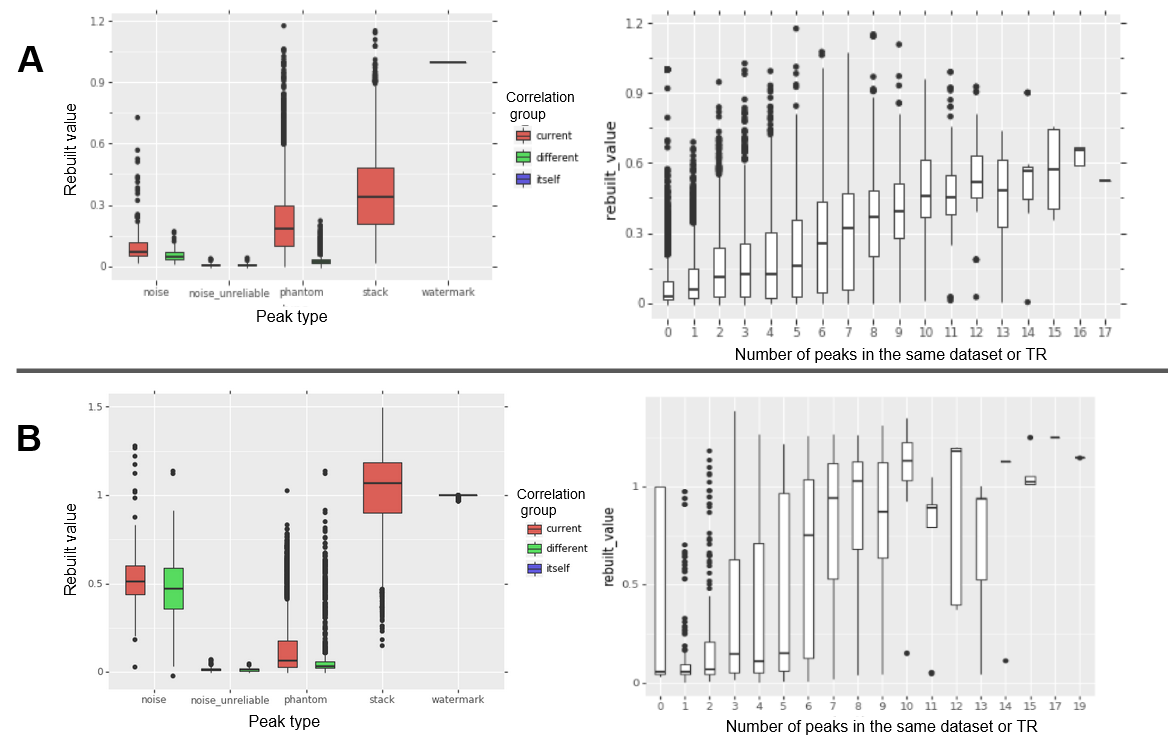

Supplement: Supplementary file 1 — Additional file 1: Fig. S1. Systematisation of artificial data analysis in 10 thousand CRM. For both a and b we use a model with deep dimension of 32, 16 convolutional filters, and LR of 1E−3. We compare a situation where such a model is too precise in b where the artificial data dimensions are 6 × 4“ with a situation in a where such a model is adequate.The left plot gives the distribution of rebuilt (max across) values of peaks depending on their type: respectively noise in reliable (R) datasets, noise in unreliable (UR) datasets, phantoms (peaks added that were not present in original matrix) and stack (peaks that were in the stack of added peaks). See Additional file 2: Fig. S2 for details. The color gives the correlation group of the peak (belonged to the same group as the group where the stack of peaks was placed for this CRM, or different). “Brothers” is the total number of peaks in same line or same column (summed). In both cases, the stack of peaks (and watermark) are correctly rebuilt, and phantoms of a high value are added in the same correlation group as the stack, but not in the other group. The correct rebuilding of the watermark shows lonely peaks can still be learned when frequent. When noise is added in the (R) datasets, it will not be part of a stack hence its usual correlators will not have been added : lacking its correlators, it atypical by our definition and gets a lower value due to this, not just because it is lonely. Noise in (UR) is discarded by the model due to its rarity. Noise scores are higher in (b) as the groups have less members, and a single noisy source represents a larger proportion of the total group learned than in (a) [file 12859_2021_4359_MOESM1_ESM.png]

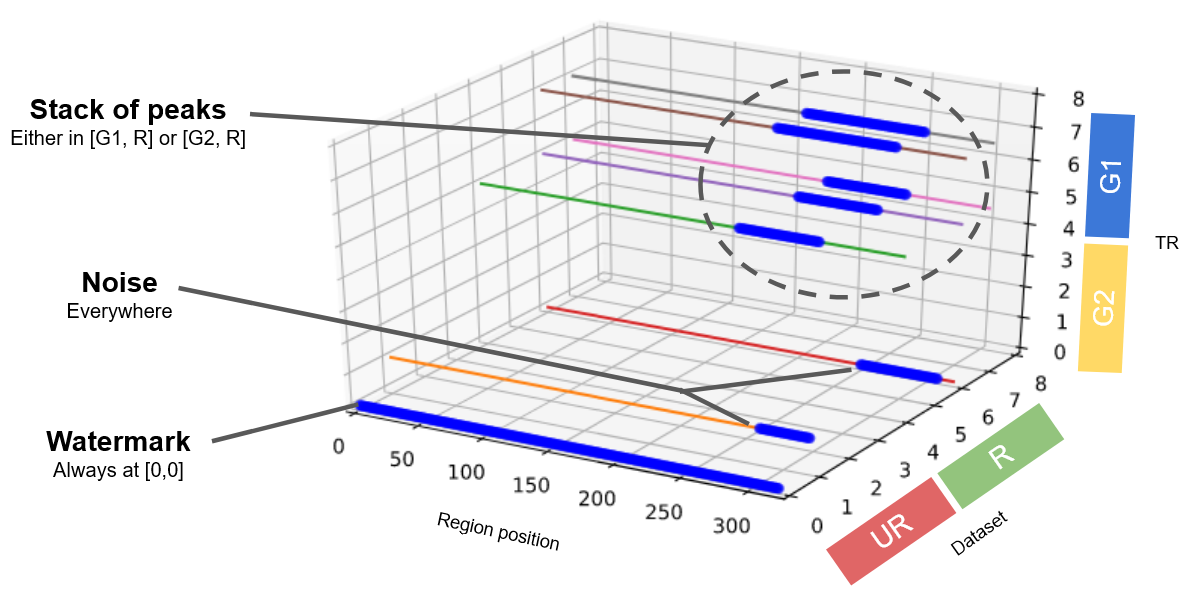

Supplement: Supplementary file 2 — Additional file 2: Fig. S2. Example of an artificially generated region representation and relevant groups, in 8 × 8 dimension. The small colored lines are only visual aids. The X axis (bottom left) is the position along the region, the Y axis (bottom right) is the dataset number and the Z axis (right) is the Transcriptional Regulator number. Datasets are split in half between “Reliable” (R) which will contain both the stack of true peaks and noise, and “Unreliable” (UR) which will contain only noise. TFs are split in the G1 and G2 groups. They can optionally be split in more than two groups. We first place a stack of peaks around a common position. These peaks will belong to either the G1 or G2 group. As a result, sources within the G1 or G2 group will correlate with each other, but will not significantly correlate with sources from outside their group. We then add noise uniformly randomly that can belong to any dataset and TR, representing anomalies. Finally a control watermark peak is added with, usually, 75% probability, representing ubiquitous TRs. All values in the tensor are 1, denoting presence. More details are available in Methods [file 12859_2021_4359_MOESM2_ESM.png]

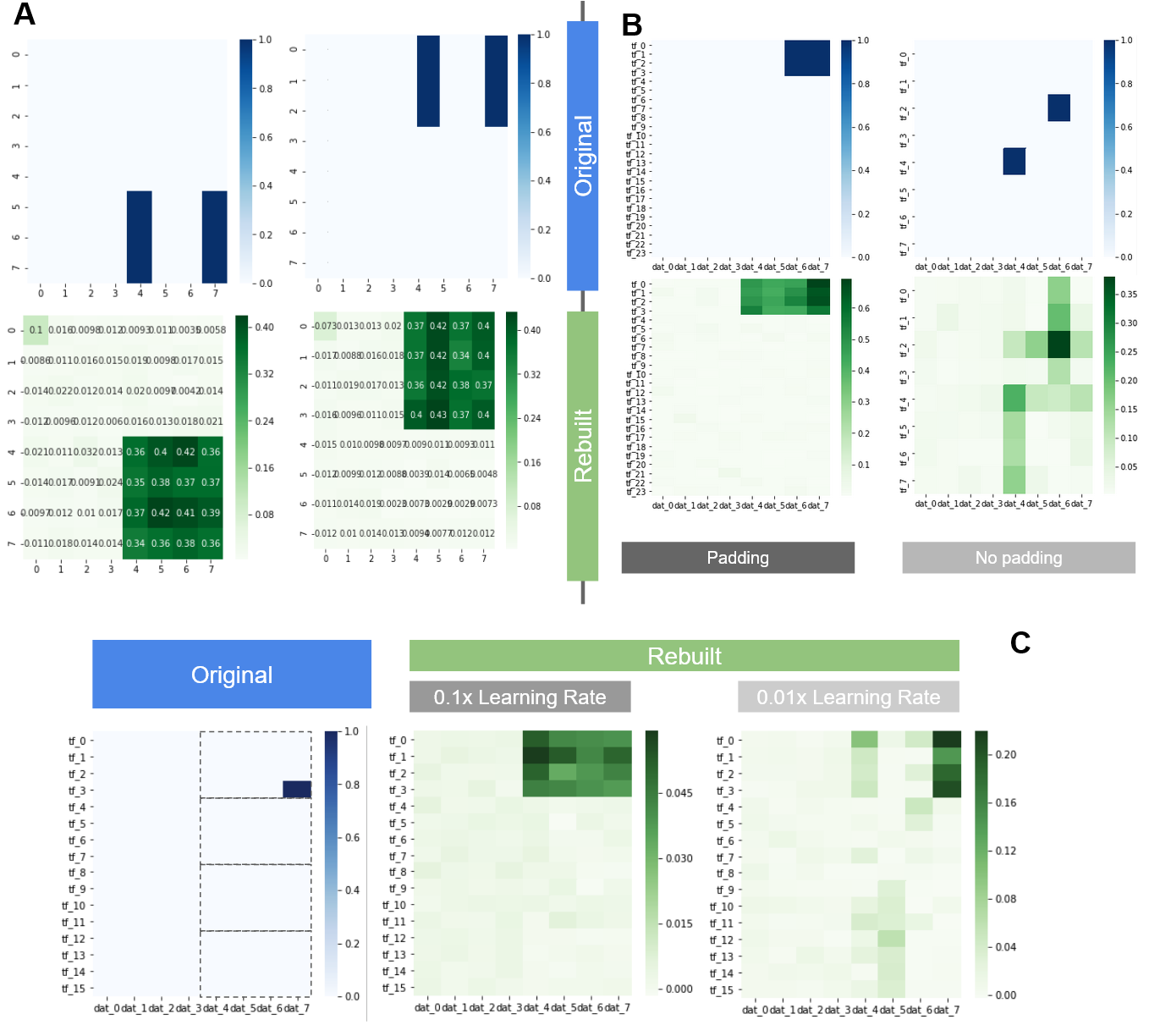

Supplement: Supplementary file 3 — Additional file 3: Fig. S3. Learning biases and budgets. a Using artificial data with group selection odds for G1 and G2 set at $${\raise0.5ex\hbox{$\scriptstyle 2$} \kern-0.1em/\kern-0.15em \lower0.25ex\hbox{$\scriptstyle 3$}}$$2/3and $${\raise0.5ex\hbox{$\scriptstyle 1$} \kern-0.1em/\kern-0.15em \lower0.25ex\hbox{$\scriptstyle 3$}}$$1/3 instead of equal. The values given in rebuilding are still only dependant on group completeness, the difference in abundance between the two groups does not influence the result. The model had 16 filters, deep dimension of 32 and LR of 1E−4. b Using two equiprobable non overlapping groups, as per Additional file 2: Fig. S2. The only difference between “padding” and “No padding” is that a padding of 12 lines (TFs) of zeroes were added to the matrices passed to the model. The model had 96 filters, 256 deep dimension, LR of 1E−4. This shows that even where there is no new information (in the left, the two groups G1 and G2 are still in the two top-rightmost 4x4 blocks), the precision is lower for the same model when the data dimensions are larger. c Model trained with artificial data, with 4 correlation groups, 64 filters and 600 deep dimension. The correlation groups predefined at data generation are reminded by the dotted lines. In spite of the very large information budget of the model, a LR of 1E−4 was not enough to reach an over-precise learning. Overprecision was achieved only with a much lower LR of 1E−5, which demonstrates that to reach increased precis [file 12859_2021_4359_MOESM3_ESM.png]

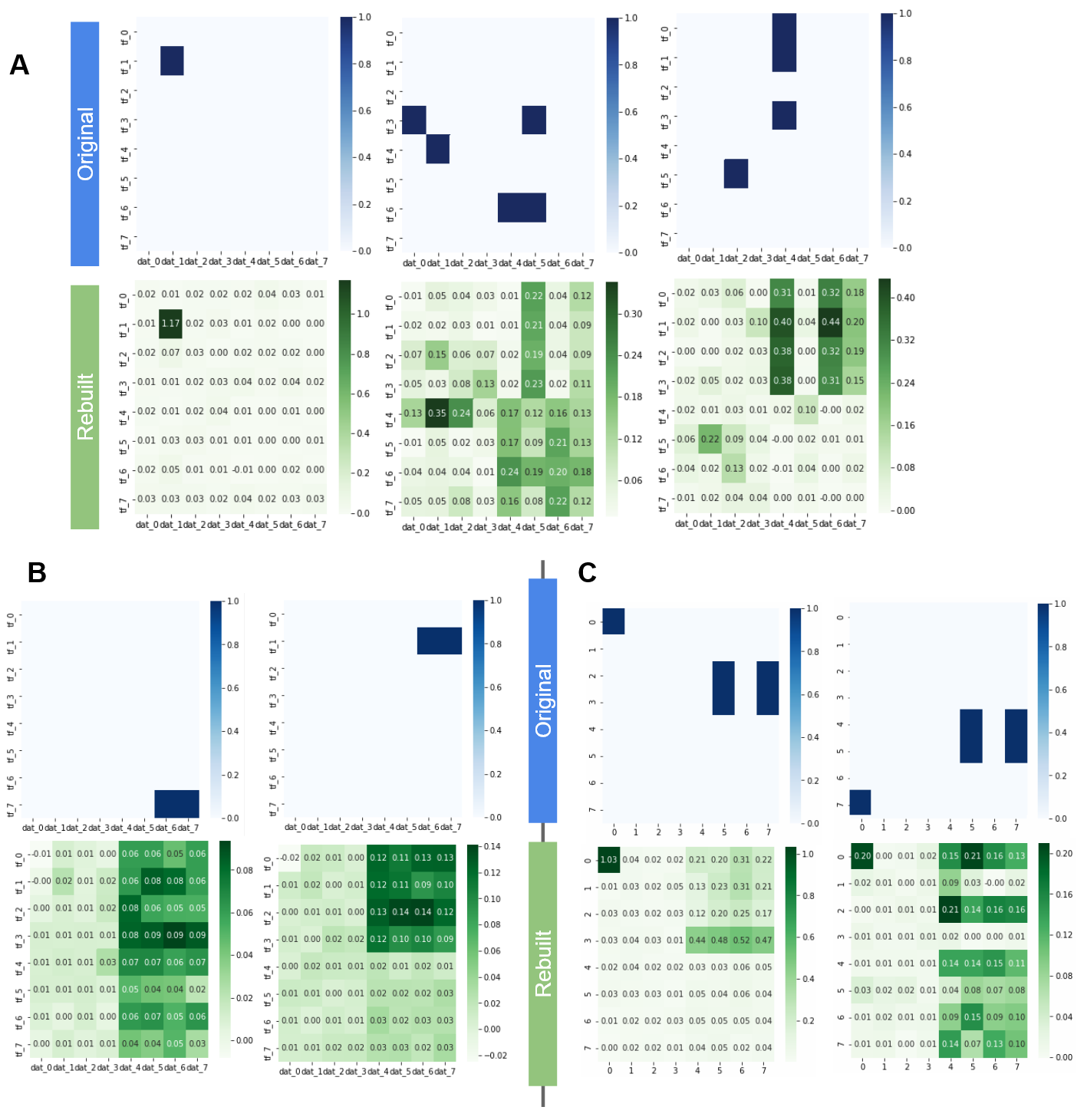

Supplement: Supplementary file 4 — Additional file 4: Fig. S4.a Usage of a weighted loss. We use a model with 24 filters, deep dimension of 64 and a LR of 1E−4. We use artificial data with the same generation process as usual, as detailed in Additional file 2: Fig. S2. However, we assign a weight of 10 in the loss to all UR datasets (0–4 included), which means that when computing the loss errors on these dimensions count 10x as much. Similar models without weighting would entirely discard peaks from suich UR datasets (Fig. 2), but now they are now learned with high precision, almost individually, while the precision is not as great for the non-weighted sources. This highlights the role of weighting in directing the learning towards specific sources and on the process of learning in general. b and c Overlapping groups. For this figure, when generating the data the two possible groups to choose from when placing the stack were not “G1” and “G2” but “G1” and “G1+G2 = all TRs”. This means the second groups overlaps with the first, and in fact contains all its sources plus its own exclusives. Both groups had 50% odds of being selected. Such overlapping groups are hard to learn and needs careful parametrization, as we explain in Methods. This requires use of 2× weighting for the sources of G2, and early stopping at 16 epochs. In B, the overlapping groups are learned properly and we see that G2 produces phantoms for G1. The model used had 16 filters and 32 deep dimension and a LR of 1E−4. c Is an example of difficulties that can be encountered. Done with a model of 24 filters, 64 deep dimension, a higher LR of 1E−3 and crucially, no weighting. The sources of G2 still produce some phantoms for the sources in G1, but those are much fainter, and the rebuilt groups are not homogeneous. Note that a lower LR of 1E−4 for this LR resulted in increased precision as would be expected, with more precise groups for G1 and no overlapping phantoms [file 12859_2021_4359_MOESM4_ESM.png]

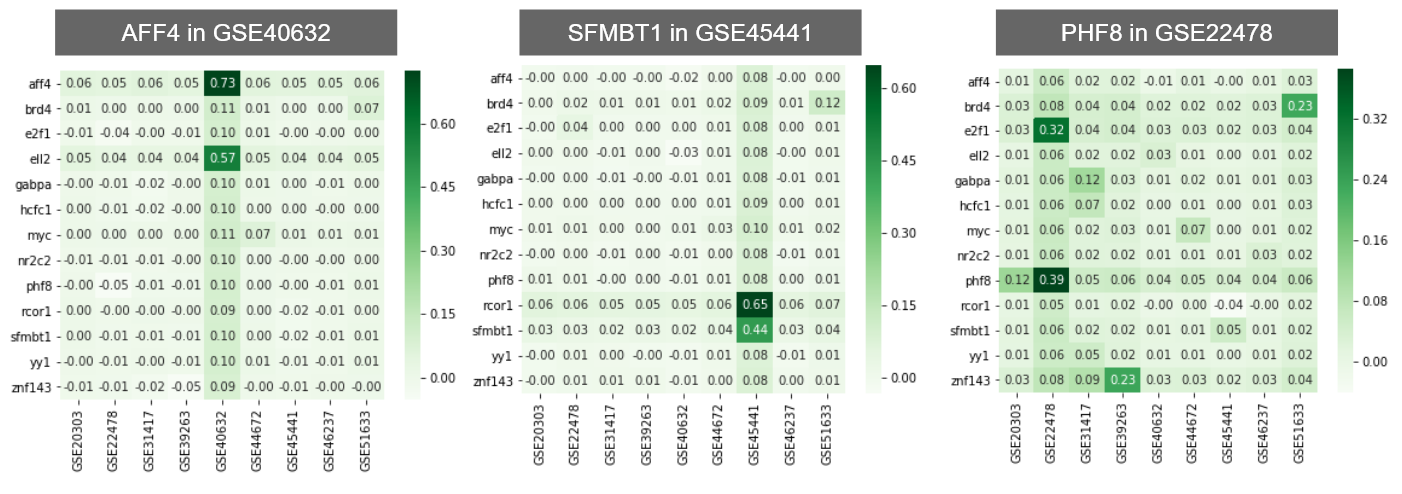

Supplement: Supplementary file 5 — Additional file 5: Fig. S11. Estimating the correlation groups certain sources belong to. This is done in the HeLa cell line, with the legend indicating respectively the Transcriptional Regulator and dataset concerned. As detailed in Methods, for each given source we create an empty CRM representation with a peak along its length for this source only, and pass it to the trained model. The result, shown above, is the sum across the X axis of the rebuilt tensor. The cross ‘+’ pattern is due to crumbing, one needs to be mindful of it when interpreting. Note that several sources (BRD4, SFMBT1) are present in more than one group [file 12859_2021_4359_MOESM5_ESM.png]

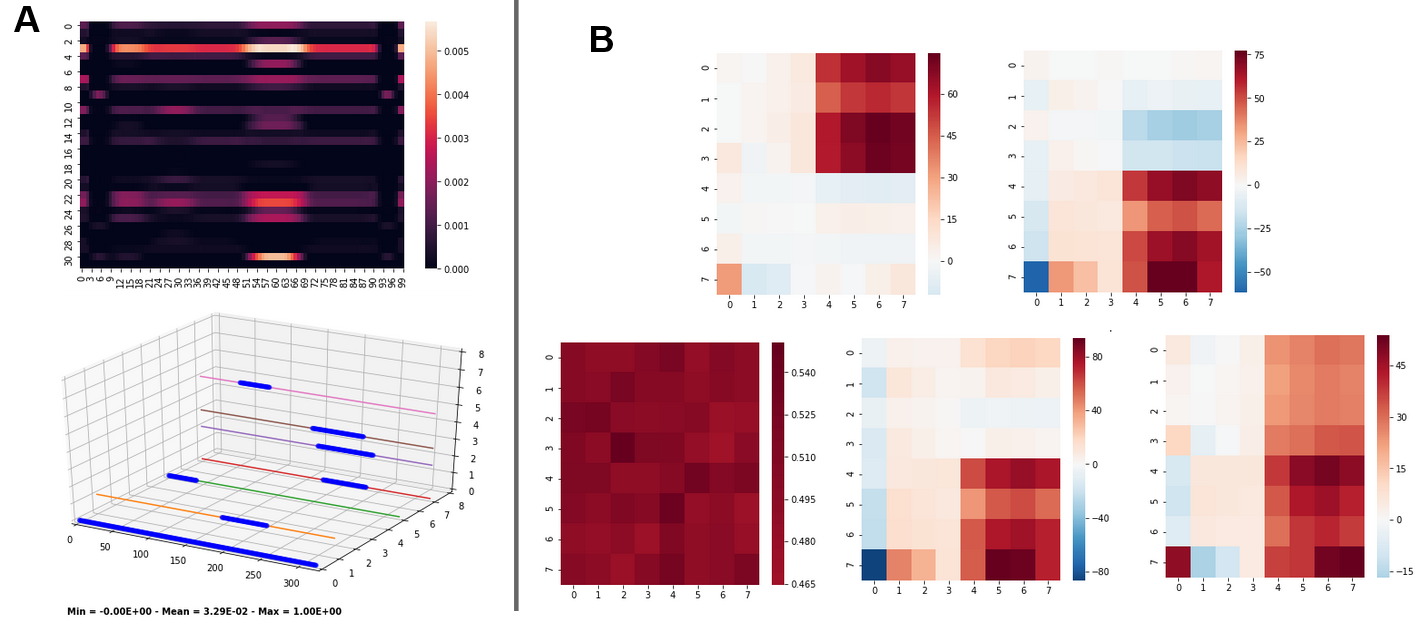

Supplement: Supplementary file 6 — Additional file 6: Fig. S6. Example of interpretability in artificial data. a The bottom figure presents an example of artificial tensor on the bottom, and the top heatmap gives the squared activation of encoded dimension when this representation is passed to the model. The parameters used are the same as Fig. 2a. b Gives several ur-examples (average across X axis) of CRMs that would maximally activate one row in the encoded dimension (rows in top left). The focus is mostly on the correlation groups as a whole. This is useful as a focus map to see where the model focuses its learning, but the final rebuilt tensor is not simply a linear combination of those, as evidenced by the fact that some ur-examples focus on both correlation groups. Some dimensions are never used, and redundancies were observed. Note that watermark is visible in those ur-examples only when it is not added 100% of the time (and therefore is a variable and not a constant) [file 12859_2021_4359_MOESM6_ESM.png]

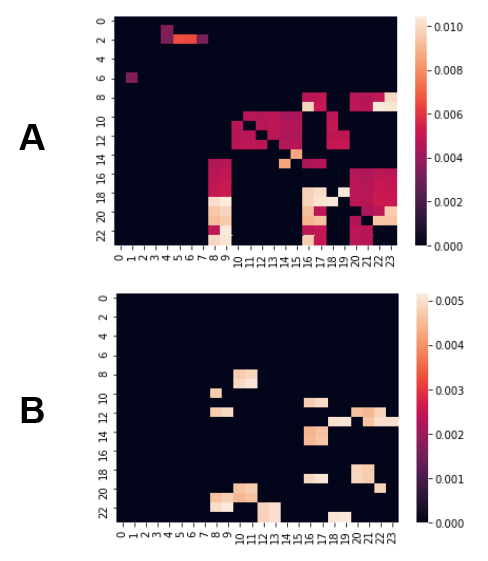

Supplement: Supplementary file 7 — Additional file 7: Fig. S5. Q-score matrices giving the contributions for each pairs of dimensions for two models from Fig. 3. Lower is better. a Corresponds to the model with 8 Deep dimension and b to the model with 256 deep dimensions. The numbers of both the X and Y axis have the same significance : 0–7 represent datasets 0 to 7, and 8–23 represent the TRs 0 to 15. The Q-score assesses, for each couple of dimensions (datasets with datasets and TRs with TRs) if the presence of one results in a higher score when present for the other, or in higher phantoms. The better model has lower Q-score, as the 8 groups were learned properly. The Q-score is currently a work in progress but is informative as to the larger trends to learning. More details are presented in Methods [file 12859_2021_4359_MOESM7_ESM.png]

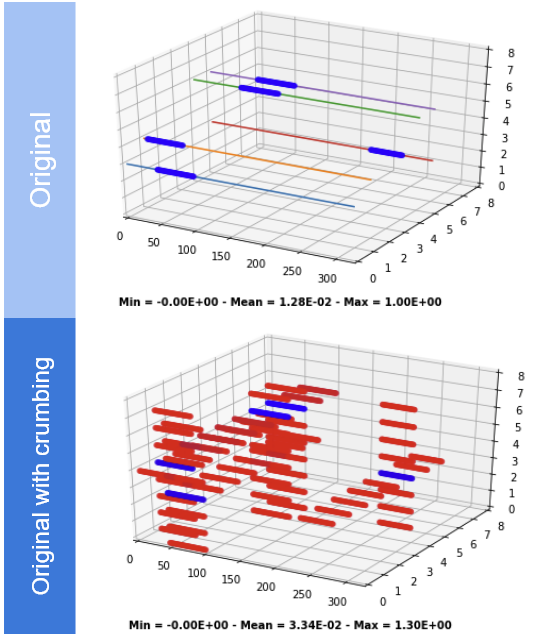

Supplement: Supplementary file 8 — Additional file 8: Fig. S9. Crumbing. On the figure, values from low to high and red to blue. Thin lines are a visual aid. Crumbing is added to real data matrices to fight sparsity. For each nonzero value in the original CRM representation at position [x,y,z], 1/10th of this value is added to all positions at [x,:,z] and [x,y,:], meaning for all datasets sharing the same TR and all TRs sharing the same dataset, forming a “+” pattern. This is necessary because on very sparse data, such as the real data tends to be, the model can easily fall in the learning trap of rebuilding a completely empty tensor [file 12859_2021_4359_MOESM8_ESM.png]

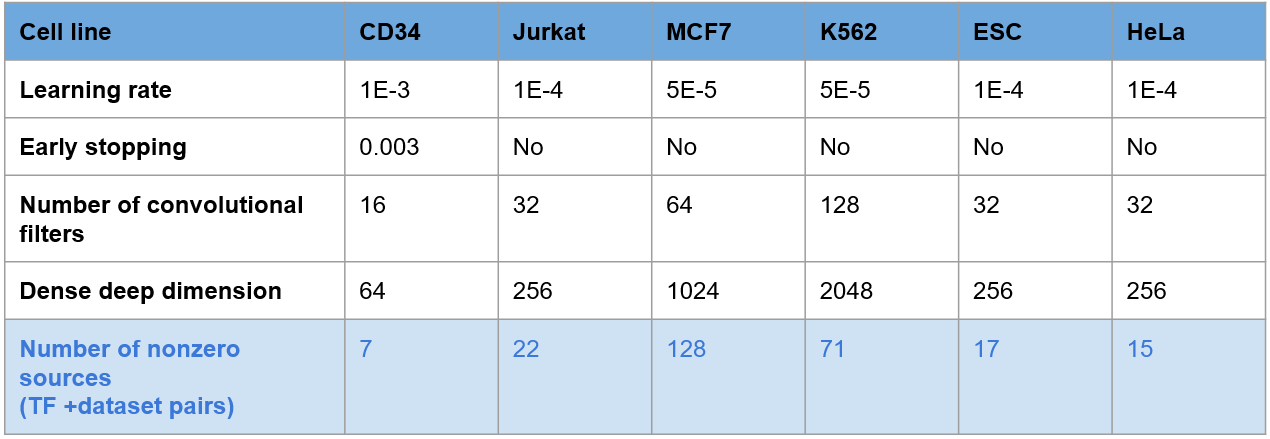

Supplement: Supplementary file 9 — Additional file 9: Table S2. Parameters used when processing real ReMap data. This provides a baseline based on the dimensions of the data for our cell lines. Dimensions provided are for comparison purposes, to provide a baseline. Other parameters (regularisation, etc.) have the same value for all cell lines, given in the Methods section in the paper. The nonzero sources gives the number of sources encountered often enough (at least one in several thousand CRMs depending on cell line) that a normalization coefficient was computed for them [file 12859_2021_4359_MOESM9_ESM.png]

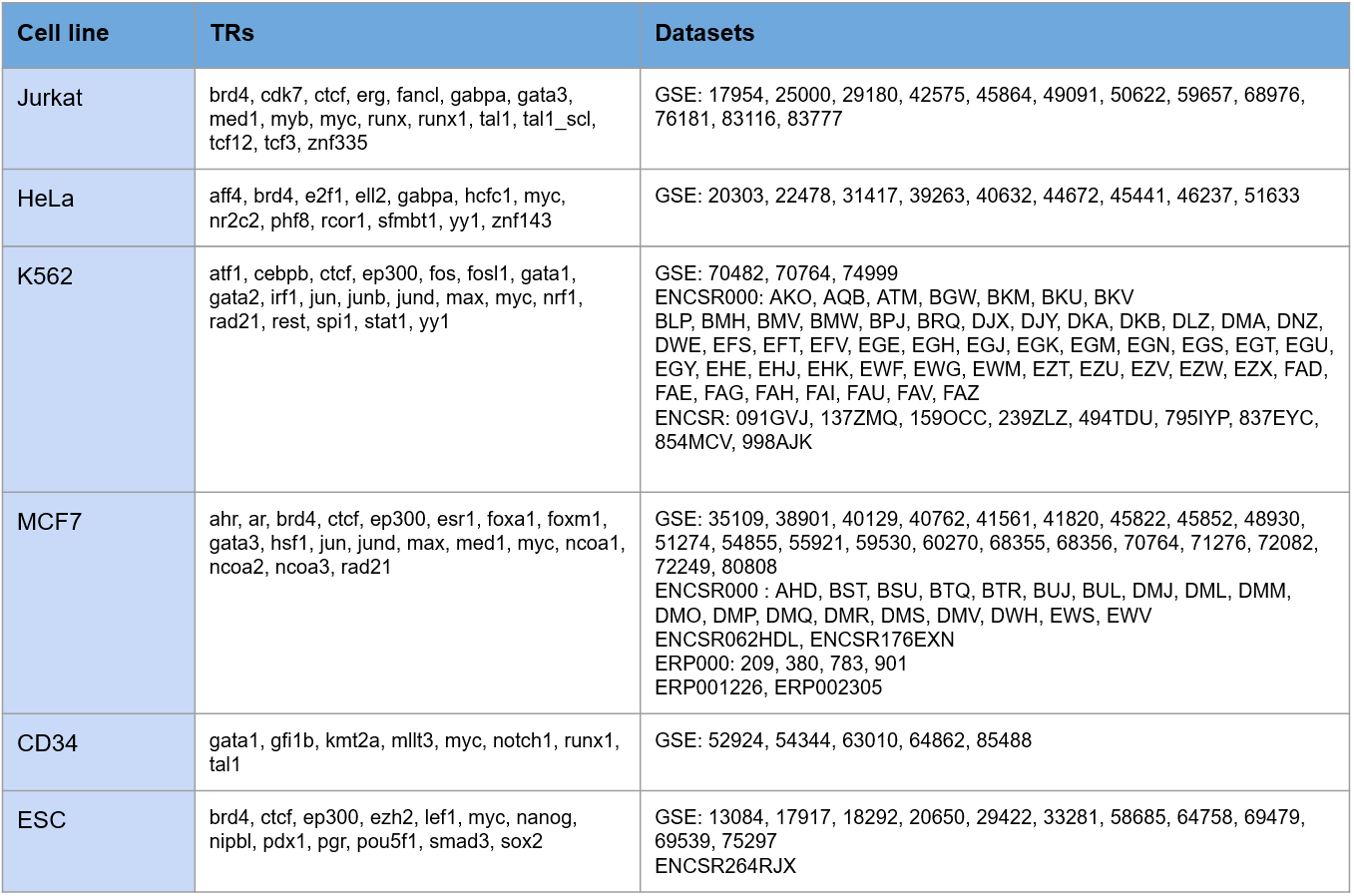

Supplement: Supplementary file 10 — Additional file 10: Table S1. List of datasets and Transcriptional Regulators from ReMap 2018 used in this study. Datasets IDs are grouped by prefix in the table: for example, “GSE52924” and “GSE54344” are grouped as “GSE: 52924, 54344”. For the cell lines, if variants are present in the ReMap data, they are not kept. For example, CD34 does not include CD34_condition1 [file 12859_2021_4359_MOESM10_ESM.png]

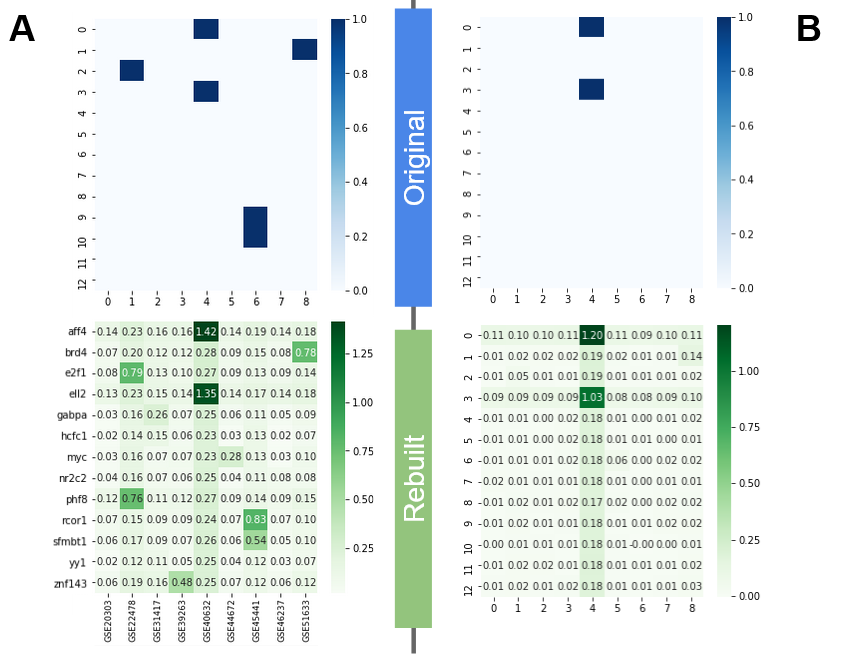

Supplement: Supplementary file 11 — Additional file 11: Fig. S8. Demonstration of overlapping groups learning by the model. a Gives a true HeLa CRM and its rebuilding, and b is the same CRM after removing all peaks excepted for those belonging AFF4 and ELL2. Even though they are a group almost by themselves (Additional file 5: Fig. S11), removing all other peaks results in AFF4 and ELL2 having a lower score in the rebuilding of A than B as BRD4 and others also contribute to their group, even though they are learned in another group. This confirms overlapping groups are possible in real data, but subject to caveats describes in Methods. Figures give the maximum across the X axis. Parameters are given in Additional file 9: Table S2. [file 12859_2021_4359_MOESM11_ESM.png]

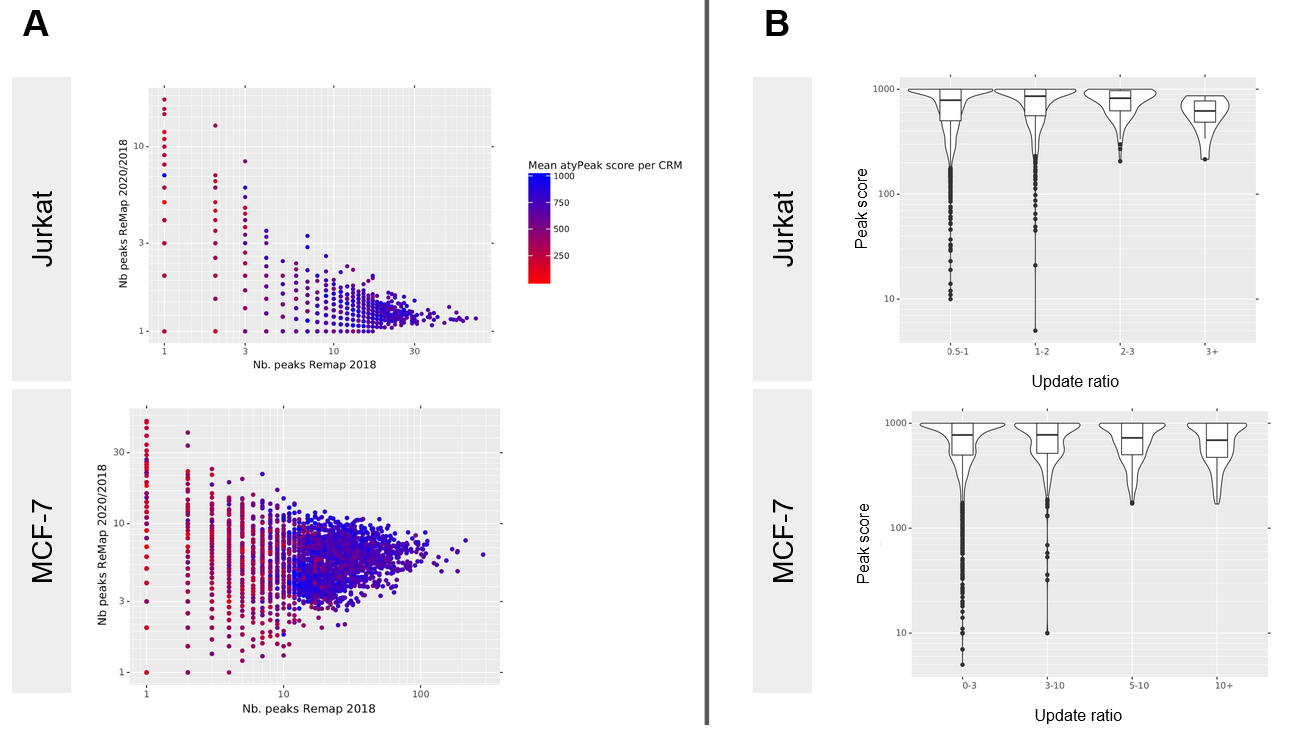

Supplement: Supplementary file 12 — Additional file 12: Fig. S7. Comparison between ReMap 2018, ReMap 2020 and predictions made by atyPeak. a For each CRM (random subselection of 10,000), number of peaks in either update and mean atyPeak score in 2018. Low scoring CRMs tend to have less peaks, and have proportionally more peaks added in 2020. b For each peak (random subselection of 5000), number of peaks in 2020 for the same TF in the same CRM divided by the number in 2018, potentially from any number of databases. Restricted to peaks in CRM with average score of at least 500 to prevent bias described in subfigure A. Log scale is used for the score to emphasize the low-scoring peaks. We see that peaks with a score of under 250 are more rarely confirmed in 2020 [file 12859_2021_4359_MOESM12_ESM.png]

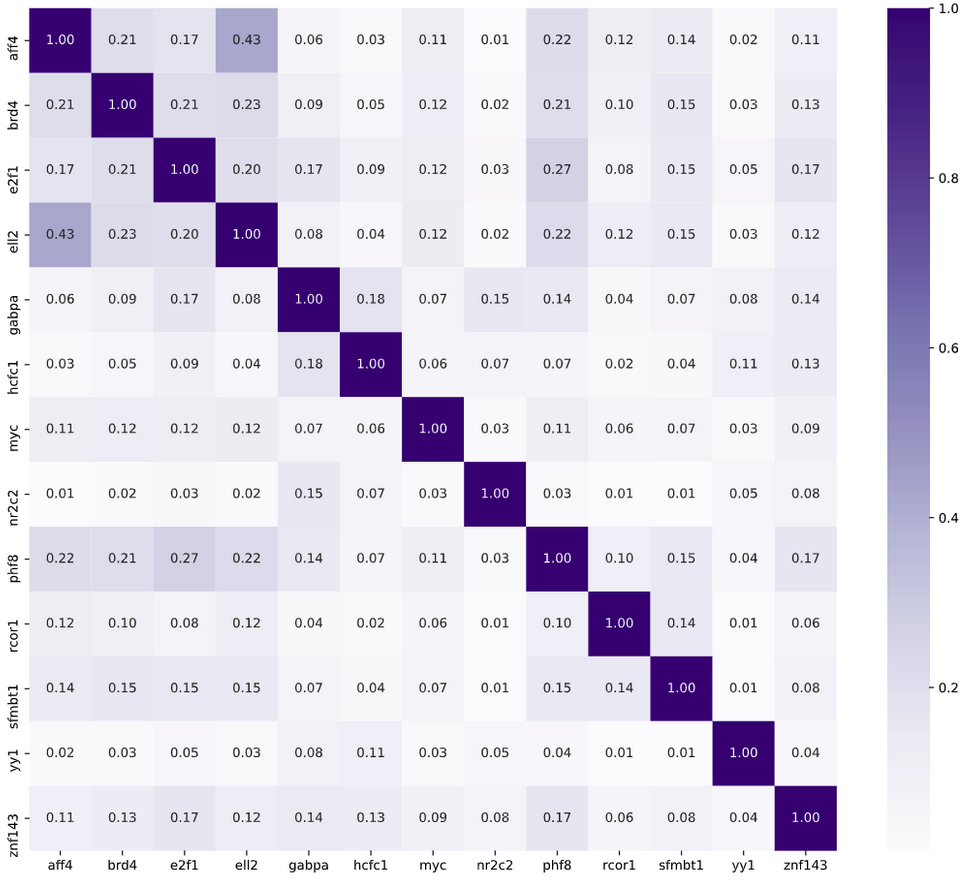

Supplement: Supplementary file 13 — Additional file 13: Fig. S12. Jaccard index A∩B/A∪B for HeLa Transcriptional Regulators. Based on the CRM representations we created for this data. Those are provided for comparison purpose and interpretation of the extracted correlations learned by the model in HeLa [file 12859_2021_4359_MOESM13_ESM.png]

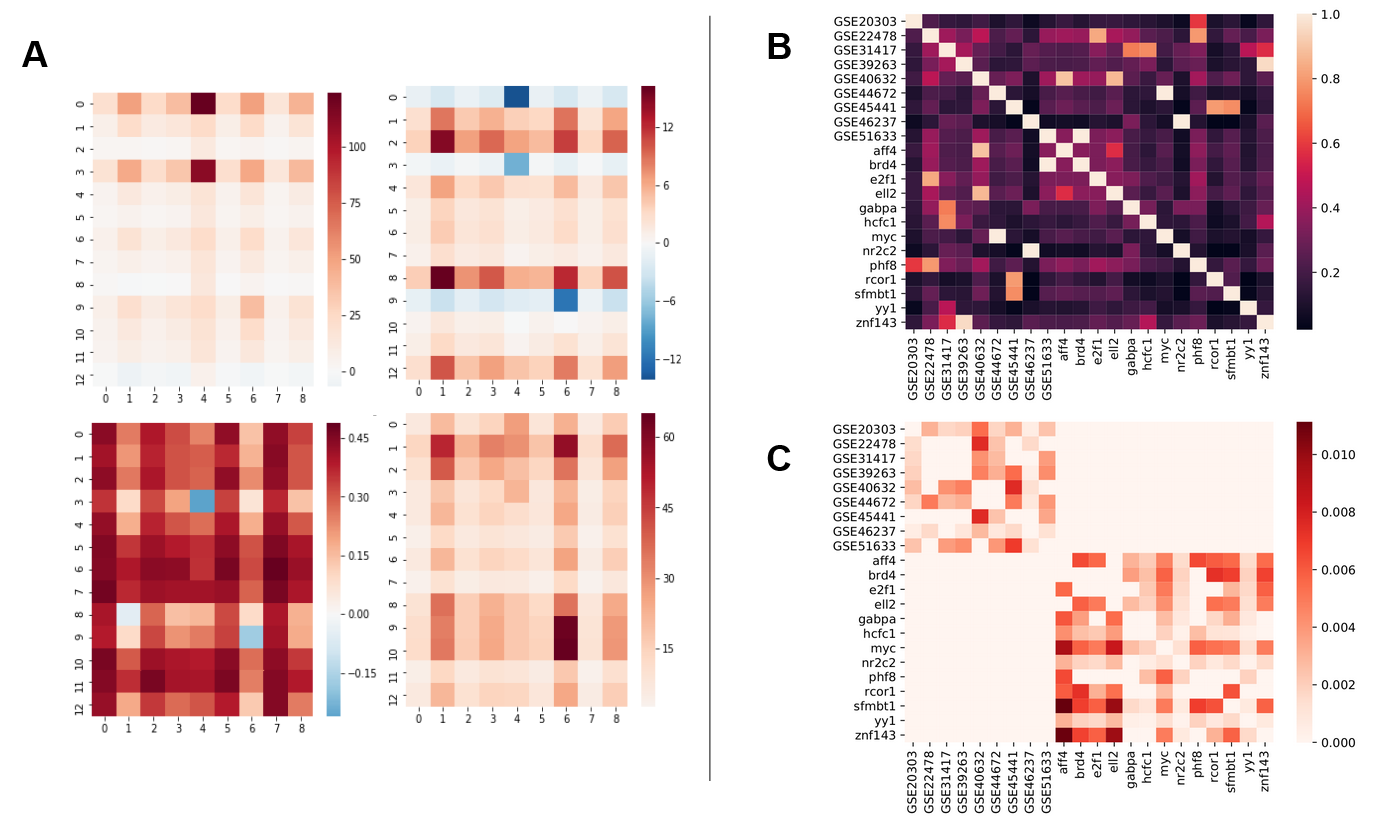

Supplement: Supplementary file 14 — Additional file 14: Fig. S10. Analysis of a trained HeLa model. a Presents some ur-examples (maximally activating CRMs for each element of the encoded dimension) summed across the X axis, calculated on a trained HeLa model. b Is HeLa correlation matrix between all dimensions like in the Q-score, cis the Q-score contributions. We observed that using a higher deep dimension can still help reach a lower loss, even with redundancies in the ur-examples, including on real data [file 12859_2021_4359_MOESM14_ESM.png]

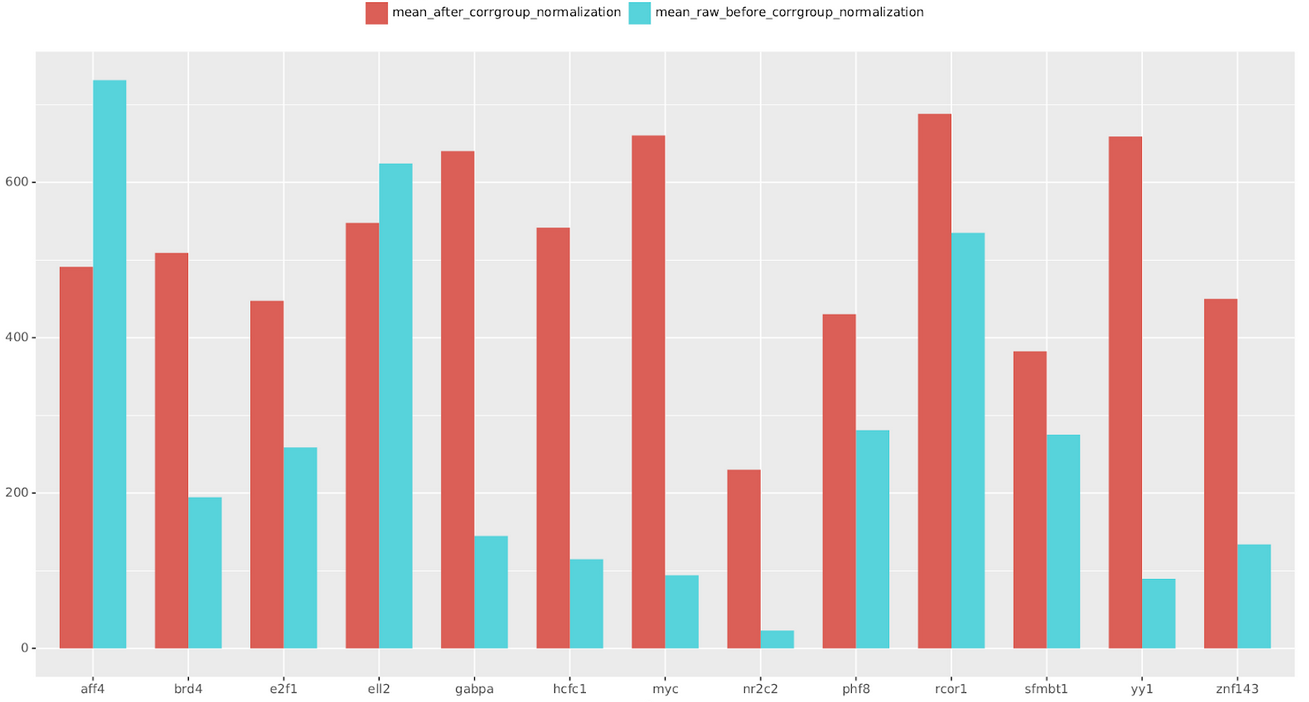

Supplement: Supplementary file 15 — Additional file 15: Fig. S13. Mean score per TR in HeLa before and after applying the normalization detailed in “Normalization of correlation group biases” in Methods, but before centering and reducing it based on the mean score for each TR. In summary, this normalization corrects biases due to average completeness differences between groups. After applying this part of our normalization, the means tend to be closer between the different TRs, correcting the various biases we detailed. For example, sources learned as part of larger groups like BRD4 (see groups in Addiotional file 5: Fig. S11) get a needed boost to their score [file 12859_2021_4359_MOESM15_ESM.png]
